# Multiparametric Single-Vesicle Flow Cytometry Resolves Extracellular Vesicle Heterogeneity and Reveals Selective Regulation of Biogenesis and Cargo Distribution

**DOI:** 10.1021/acsnano.3c11561

**Published:** 2024-04-05

**Authors:** Ariana K. von Lersner, Fabiane Fernandes, Patricia Midori Murobushi Ozawa, Marques Jackson, Matthieu Masureel, Hoangdung Ho, Sierra M. Lima, Tatyana Vagner, Bong Hwan Sung, Mohamed Wehbe, Kai Franze, Heather Pua, John T. Wilson, Jonathan M. Irish, Alissa M. Weaver, Dolores Di Vizio, Andries Zijlstra

**Affiliations:** 1 Program in Cancer Biology, Vanderbilt University, Nashville, Tennessee 37232, United States; 2 Department of Pathology, Microbiology and Immunology, Vanderbilt University Medical Center, Nashville, Tennessee 37232, United States; 3 Institute of Applied Biosciences and Chemistry, Hogeschool Arnhem en Nijmegen University of Applied Sciences, Nijmegen 6525 EM, Gelderland, Netherlands; 4 The Center for EV Research, Vanderbilt University, Nashville, Tennessee 37232, United States; 5 Department of Cell and Developmental Biology, Vanderbilt University School of Medicine, Nashville, Tennessee 37232, United States; 6 Department of Research Pathology, Genentech, San Francisco, California 94080, United States; 7 Department of Structural Biology, Genentech, San Francisco, California 94080, United States; 8 Department of Surgery, Cedars-Sinai Medical Center, Los Angeles, California 90048, United States; 9 Department of Chemical and Biomolecular Engineering, Vanderbilt University, Nashville, Tennessee 37232, United States; 10 KNIME GmbH, Konstanz 78467, Germany

**Keywords:** extracellular vesicle, heterogeneity, biogenesis, multiplex, multiparametric, dimensional reduction, flow cytometry

## Abstract

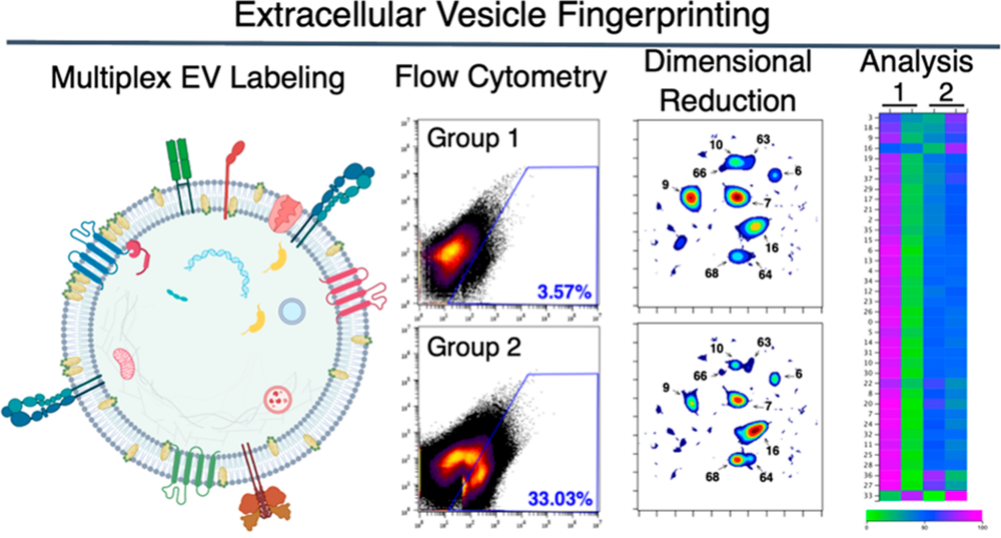

Mammalian cells release a heterogeneous array of extracellular vesicles (EVs) that contribute to intercellular communication by means of the cargo that they carry. To resolve EV heterogeneity and determine if cargo is partitioned into select EV populations, we developed a method named “EV Fingerprinting” that discerns distinct vesicle populations using dimensional reduction of multiparametric data collected by quantitative single-EV flow cytometry. EV populations were found to be discernible by a combination of membrane order and EV size, both of which were obtained through multiparametric analysis of fluorescent features from the lipophilic dye Di-8-ANEPPS incorporated into the lipid bilayer. Molecular perturbation of EV secretion and biogenesis through respective ablation of the small GTPase Rab27a and overexpression of the EV-associated tetraspanin CD63 revealed distinct and selective alterations in EV populations, as well as cargo distribution. While Rab27a disproportionately affects all small EV populations with high membrane order, the overexpression of CD63 selectively increased the production of one small EV population of intermediate membrane order. Multiplexing experiments subsequently revealed that EV cargos have a distinct, nonrandom distribution with CD63 and CD81 selectively partitioning into smaller vs larger EVs, respectively. These studies not only present a method to probe EV biogenesis but also reveal how the selective partitioning of cargo contributes to EV heterogeneity.

Extracellular vesicles (EVs) are membrane-partitioned particles which contain biologically active cargo and functionally contribute to intercellular communication in health and disease.^1^ The detection and characterization of these vesicles is therefore thought to be a means to derive biological insights and attain biomarkers that inform on the status of a patient. Although frequently referred to as a single class cumulatively defined as lipid bilayer-enclosed particles, the term “extracellular vesicles” encompasses a heterogeneous group of particles produced by multiple biogenesis pathways which vary greatly in both size and composition.^2^ EVs are commonly characterized by their size.^3^ While imperfect, this metric is informative in distinguishing between smaller EVs (S-EVs) ranging from 30 to 200 nm and larger EVs (L-EVs) ranging from 200 to >1000 nm.^4^ Given that EVs have been demonstrated to convey a diverse array of biological activity,^5^ the heterogeneity of EV populations suggests that unique populations of EVs convey distinct functions. This is underscored by studies evaluating the functional role of S-EVs vs. L-EVs.^6−8^ Moreover, changes in EV heterogeneity can reflect a change in biological state (i.e., cancer vs normal) thereby serving as a clinically informative biomarker.^9,10^

While much remains unknown about EV biogenesis, it has been established that they are generated through at least two distinct mechanisms (Figure 1a). Cells can produce EVs through exocytosis of endosome-derived multivesicular bodies (MVBs) that fuse with the plasma membrane to release exosomes into the extracellular space.^2,3^ This biogenesis pathway is regulated by the small GTPase Rab27a, which controls docking of MVBs with the plasma membrane.^11^ These EVs are thought to be ≤200 nm. Alternatively, EVs can arise through direct budding and fission of the plasma membrane (ectocytosis) to produce ectosomes.^2,3^ These vesicles range broadly in size and can be as small as the exosomes, but also significantly larger (≥1 μm).^4,12^ Not only do EVs vary in size, they are also known to vary in composition based on the incorporation of cargo (lipids, proteins, carbohydrates, and nucleic acids) during biogenesis.^13−16^

**Figure 1:**
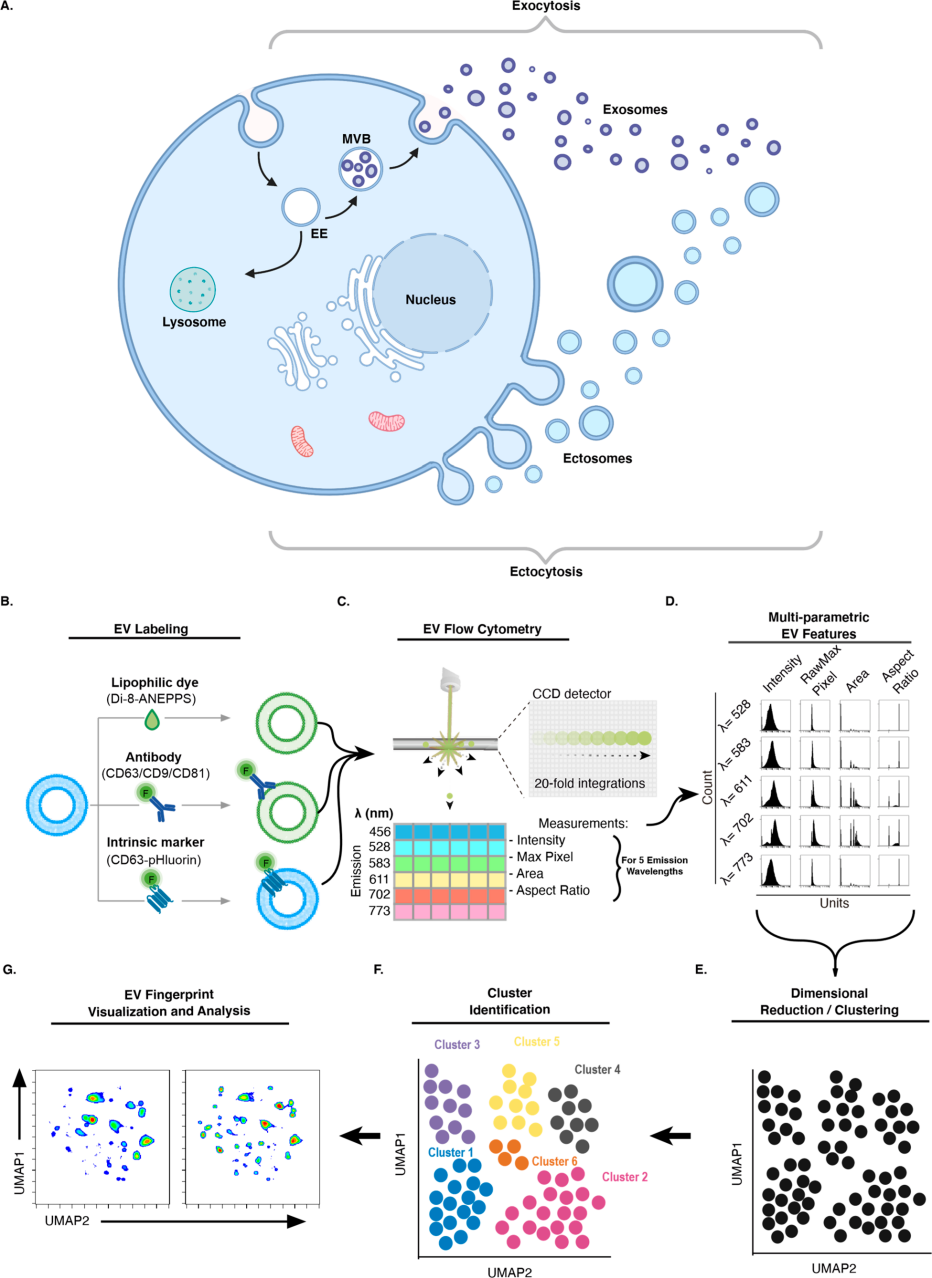
EV Fingerprinting overview. (A) Schematic representation of EV biogenesis through exocytosis and ectocytosis leading to the formation of exosomes and ectosomes, respectively. Created with BioRender.com. “EE” early endosome, “MVB” multivesicular body. (B) Extrinsic labeling of EVs using the lipophilic dye, di-8-ANEPPS (di8), and fluorescently conjugated antibody or intrinsic labeling using fluorescent fusion proteins (pHluorin_M153R tagged CD63). (C) Optical triggering on the CellStream uses a time delay integration charge-coupled device (TDI-CCD) to collect measurements (including Intensity, RawMax Pixel, Area, and Aspect Ratio) simultaneously for up to six emission wavelengths (em = 456, 528, 583, 611, 702, and 773). (D) A matrix histogram of measurements for the five emission wavelengths collected for di8 excited at 488 nm demonstrates the multiparametric nature of EV features collected from this lipophilic, environment sensitive dye. (E) Dimensional reduction embedding and clustering of multiparametric EV features using UMAP. (F) Cluster identification was done using HDBSCAN. (G) Representative results of a UMAP density plot comparing two paired samples.

EVs have been shown to be selectively enriched for certain cargo such as the tetraspanins (TSPANs) CD9, CD63 and CD81.^17^ While protein and nucleotide cargo of EVs are intensely studied, less is known about their lipid composition and how this contributes to EV heterogeneity.^18,19^ While biological membranes are frequently represented as homogeneous layers, they are, in fact, highly organized structures that display lateral phase separation and lipid domain segregation.^20,21^ Membrane partitioning is achieved in part by the organization of the membranes into a liquid ordered (Lo) or disordered (Ld) state.^21−25^ This organization is functionally important for complex activities, such as signal transduction^26^ membrane trafficking^27,28^ and enzyme activity.^29^ Moreover, it contributes to membrane shape^20,30^ and fusogenicity.^24,31,32^ Membrane order is influenced by many factors including fatty acid chain saturation (e.g., unsaturated vs saturated phospholipids) and lipid packing (e.g., the inclusion of cholesterol), which can be modeled with multicomponent bilayers in synthetic liposomes created with controlled lipid composition.^21,23,25,33^ Bulk lipidomics of EVs has demonstrated that they vary in lipid composition, however, such variation at the single EV level has not been evaluated previously.^34−36^

Single-EV flow cytometry methods are experiencing increased adoption given their ability to characterize molecular cargos on a single vesicle basis. While most conventional flow cytometers are not set up to accurately detect particles smaller than 500 nm in diameter, instrumentation can be adapted to enable small particle detection.^37^ For example, Stoner et al.^38^ built a customized high-sensitive flow cytometer to extend the limit of detection to 70–80 nm. In addition, fluorescence triggering with lipophilic dyes such as with di8 demonstrated reproducible detection of EVs.^38^ Other specialized single-EV flow cytometry platforms achieve increased instrument sensitivity, in part by slowing down the flow rate which, in turn, greatly limits throughput (10,000–12,000 events/min).^39,40^ Conversely, technologies with faster flow rates (≥100,000 events/min) not only suffer from lower sensitivity, but are also susceptible to the so-called swarm effect, in which groups of small particles are registered as single large particles.^41,42^ To achieve accurate high-throughput detection of small particles at a fast flow rate (∼100,000 events/min), the Amnis CellStream platform utilizes a Time Delay Integration Charge-Coupled Device (TDI-CCD). The detection of photons across spatially separated pixels permits the capture of multiparametric image-like features, providing additional data dimensions. We exploited the gain in detection sensitivity with the pixel-based image features to resolve the EV heterogeneity.

In this study we used the uptake of lipophilic environment-sensitive membrane probes (such as di-8-ANEPPS (di8)) by the lipid bilayer, together with analysis of additional data dimensions collected by a TDI-CCD of the CellStream, to generate a single-EV flow cytometry method in which: (1) the uptake of dye was used to detect EVs and assess their relative size; and (2) the shift in emission maxima of these dyes in response to membrane order along with image parameters was used to deconvolve heterogeneous EV populations.^43−46^ We coupled these data dimensions with dimensional reduction to develop the “EV Fingerprinting” approach that deconvolves the complexity of EV populations. The method was established with highly purified EVs and validated with synthetic liposome standards of highly ordered (Lo) and disordered (Ld) membranes. Using the guidelines from the International Society for Extracellular Vesicles (ISEV) for the minimal information for studies of EVs (MISEV),^47−49^ orthogonal EV characterization methods were used to confirm EV Fingerprinting observations. EV Fingerprinting was subsequently implemented to reveal that mammalian cells produce distinct EV populations that (i) are discernible using multiparametric features derived from EV size and membrane order, (ii) are selectively and differentially impacted by disruption of exosome secretion with knockdown (KD) of Rab27a, and the overexpression of EV cargo (CD63), and (iii) carry a distinct, nonrandom distribution of cargo that can be revealed by multiplexing.

## Results

### Principle of EV Fingerprinting and Workflow

In order to deconvolve the heterogeneity of EVs, we developed a method using single-EV detection of fluorescently labeled EVs by flow cytometry, followed by dimensional reduction of multiparametric features to resolve individual EV populations (Figure 1b–g). The approach leverages changes in fluorescence intensity and emission spectra of the lipophilic dye di8, associated with variations in particle size and lipid composition to detect, discern, and quantify distinct EV populations. In addition, single-EV cargoes are visualized using a genetically encoded fluorescent marker (e.g., pHluorin-CD63)^50^ or a cargo-specific antibody (e.g., anti-CD63 antibody, Figure 1b). Fluorescently labeled EVs are detected in the Amnis CellStream flow cytometer, where 20-fold signal integration across the TDI-CCD detector enables sensitive detection of passing particles (Figure 1c). Multiparametric data of di8 staining are extracted from the final integration using four optical features across five emission wavelengths (Figure 1d, Table S1 and S2). Data reduction using Uniform Manifold Approximation Projection (UMAP) of the resulting 20 dimensions creates a 2D representation of the EV populations present in the sample (Figure 1e). Hierarchical Density-Based Spatial Clustering of Applications with Noise (HDBSCAN) is subsequently used for cluster identification (Figure 1f).^51−54^ The resulting pattern of clusters is a “Fingerprint” of distinct EV populations. Unlike previously established bulk EV analytical methods and conventional flow cytometry, EV Fingerprinting makes it possible to perform quantitative assessment of distinct EV populations and determine how they are altered by experimental manipulation, molecular perturbation, or a disease state (Figure 1g).

### Optimization of EV Detection by Fluorescent Triggering

Single-EV detection was achieved by fluorescence-triggered flow cytometry (Amnis CellStream) with di8-stained EVs by using a TDI-CCD detector to collect optical measures upon fluorescence excitation by a 488 nm laser. Two staining methods were optimized to enable analysis of samples of both low and high particle concentrations (methods #1 and #2 in Figure S1a and 1b, respectively). For samples with low EV concentrations (<1 × 10^8^/mL), including unpurified, EV-containing fluids such as conditioned medium (CM), the sample is stained with a low concentration of di8 (0.25 μM) and analyzed directly by flow cytometry. For high EV concentration (≥1 × 10^8^/mL) such as EVs purified by ultracentrifugation (UC), the sample is stained with a high concentration of di8 (2 μM) and subsequently diluted 200-fold before analysis by flow cytometry. These dye concentrations reduce background fluorescence and prevent self-quenching while serial dilution avoids swarming the detector with a high number of particles.^55,56^ In addition, method #2 is amendable to single and multiplex antibody staining of di8-labeled EVs.^57,58^ Samples are always serially diluted to ensure detection within the quantitative range of the assay. To identify the optimal staining conditions, a series of trials was completed to evaluate the impact of: (1) di8 concentration (Figure S1a–d), (2) the staining temperature (Figure S1d), (3) and the laser excitation intensity (Figure S1e,f). Based on observations from these trials, we defined the optimal staining conditions, as summarized in Table S3.

Using Method #1, we tested EV detection across a serial dilution of di8 from 4 to 0.0078 μM staining at room temperature (RT, approximately 22°C). Consistent EV detection was observed in a range from 0.0156 to 0.25 μM (Figure S1c, 100 K EV-blue) with minimal signal derived from free dye (Figure S1c, Buffer-black).

While lipophilic labeling is typically performed at RT, antibody staining for flow cytometry is often optimized at different temperatures. To assess the impact of temperature on vesicle staining, Method #2 was evaluated at RT and 37°C using 2-fold dilutions from 4 to 0.5 μM. EV detection was consistent for both temperatures at 2 μM di8 (Figure S1b and d).

The impact of excitation energy on EV detection was assessed using a 488 nm laser titration for both Methods #1 and #2. EV detection plateaued at laser powers greater than 45% for both staining methods (Figure S1e,f). Moreover, background particle detection in the absence of EVs increased when laser power was increased beyond 25% with both staining methods (Figure S1e,f). The increase in non-EV background particles adversely impacted samples with low EV count and (Figure S1e,f: 100 K 1×), we therefore used 25% laser power for subsequent assays.

### Quantitative Single-EV Detection

Single-EV detection in a flow cytometer can require extensive dilution of the source material to avoid swarming of the detector with multiple particles. However, the resulting reduction in molecular density is known to impact both microparticles and macromolecules by promoting nonspecific interactions.^59^ To offset the diminution in protein and other buffering components caused by sample dilution,^59^ we tested the benefit of molecular crowding (MC) with dextran (*M*_r_ = ∼100,000). Final dextran concentrations of 1.625%, 3.25%, and 6.5% compared to PBS (0% Dextran) improved single particle detection for both FITC-labeled nanosized beads as well as UC purified di8-stained EVs by 100–400% while increasing dextran concentration to 10% eliminated gains in detection (Figure S2a,b, respectively). In subsequent assays, dextran samples were prepared at 6.5% and diluted to 3.25% final concentration as the optimal condition for particle detection (Figure S2c,d).

Single particle detection was verified using nonfluorescent and fluorescent synthetic bead standards as well as UC purified, di8-stained EVs (Figure S2c,d). FITC-labeled sizing beads (0.5, 0.2, and 0.1 μm, respectively) were serially diluted, and the corresponding linear detection was successfully quantified (Figure S2c). To define the quantitative range of EV detection, 100,000 UC purified EVs stained by Method #2 were evaluated across a serially diluted sample (Figure S2d). The quantitative range of EV detection, where the EV count corresponded linearly to the dilution factor and the median fluorescence intensity remained consistent (MFI, Median 488–611), was identified at 50,000–400,000 count/min run time which equates to 1.5–12 × 10^7^ EV/mL when correcting for the volume analyzed (gray box, Figure S2d). The specificity of lipid-based vesicle detection was confirmed through detergent lysis of the EVs. Indeed, the detection of di8-positive (di8+) particles was abrogated upon lysis with 0.5% NP40 detergent, confirming specificity of EV detection (Figure S2e).

**Figure 2:**
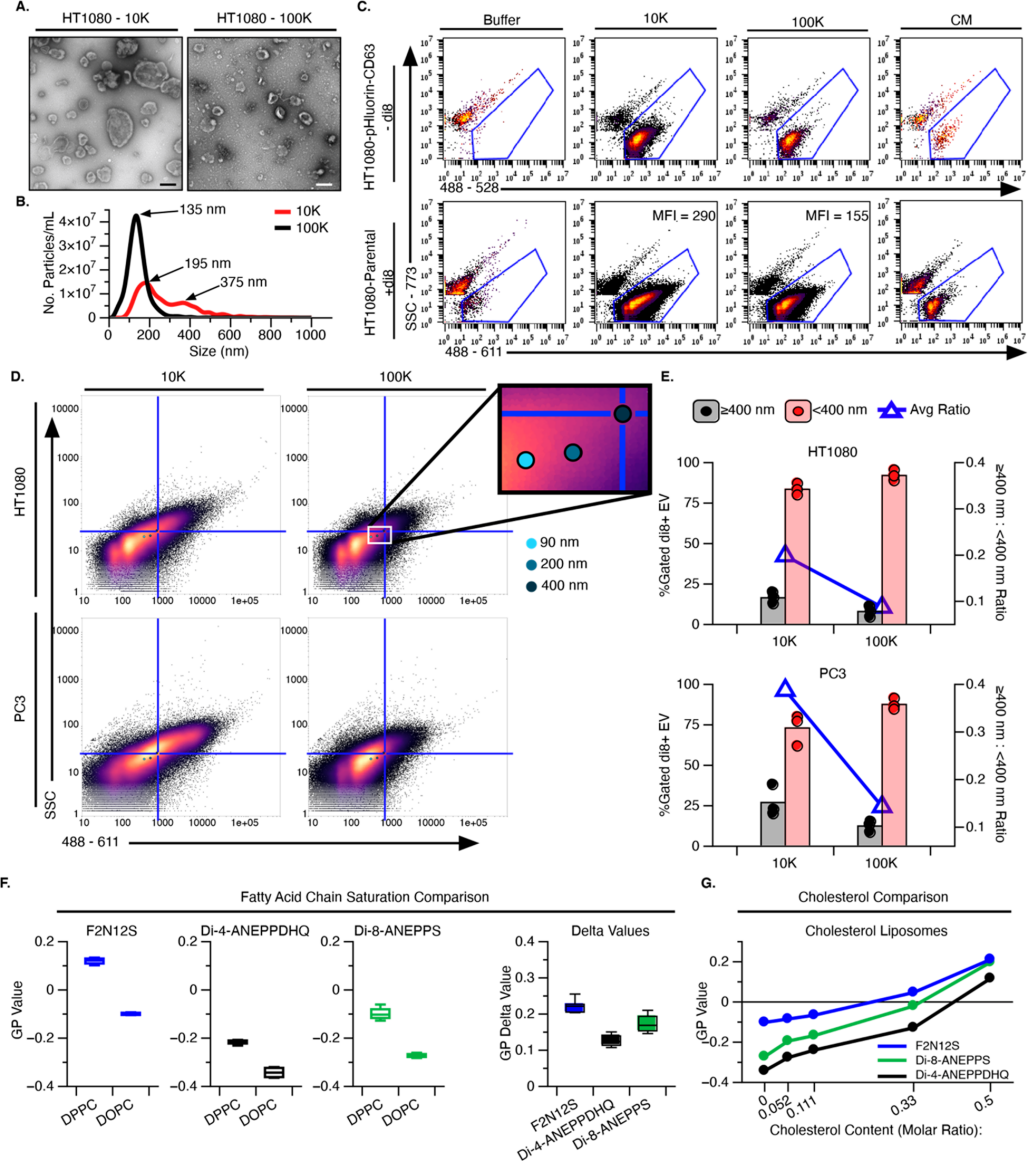
Quantitative detection of single EVs by flow cytometry. (A) Negative staining of transmission electron micrographs (TEM) of 10 K (left) and 100 K (right) UC purified EV preps from HT1080 parental cells. Scale bar: 200 nm. (B) Representative traces from nanoparticle tracking analysis (NTA) of 10 K and 100 K EV preparations. (C) Representative flow cytometry scatter plots of intrinsically labeled HT1080-pHluorin-CD63 (top) and extrinsically di-8-ANEPPS (di8) stained HT1080-Parental EV (bottom) samples from 10 K and 100 K DG-UC EV preparations and conditioned media (CM). Gating of the EVs was performed against the buffer controls. (D) Scatter plots of EVs in 10 K and 100 K DG-UC preparations from HT1080 and PC3 cells. The median scatter (SSC) and 488–611 (excitation–emission) MFI of sizing liposomes (90, 200, and 400 nm) is shown within the scatter plots, as indicated (see also Figure S3). Sizing liposomes were measured in triplicate with the 400 nm liposome used as the midpoint for quadrant gates. The upper right quadrant represents EVs ≥ 400 nm. (E) Relative abundance of EVs < 400 nm (pink) or ≥ 400 nm (gray) in isolated EVs from HT1080 and PC3 cells. Bar plots represent quantitation of the dilution-corrected number of EVs (left *y*-axis) and the ratio of larger to smaller EVs (blue triangle, right *y*-axis. (F,G) Evaluating the ability of flow cytometry to detect membrane order with the lipophilic, environment sensitive dyes F2N12S (blue), Di-4-ANEPPDHQ (black), and Di-8-ANEPPS (green) by changing fatty acid chain saturation of phospholipids (DPPC vs DOPC) (F) or incorporating cholesterol (DOPC) (G). Average GP values from (*n* = 4) technical replicates for each condition.

The validated EV flow cytometry strategy was subsequently applied to EVs purified from fibrosarcoma cells (HT1080)-CM by a standard sequential UC method^60^ designed to enrich for L-EVs at 10,000*g* (10 K) and for S-EVs at 100,000*g* (100 K) (Figure 2). Transmission Electron Microscopy (TEM) confirmed the expected size-selective enrichment in the two respective preparations (Figure 2a, scale bar = 200 nm). In parallel, nanoparticle tracking analysis (NTA) confirmed the differential enrichment for larger EVs in the 10 K UC prep and for smaller EVs in the 100 K UC prep (Figure 2b). Flow cytometry successfully detected both intrinsically labeled EVs (pHluorin-CD63) and extrinsically labeled EVs (di8) in the UC purified EV preparations (10 and 100 K, Figure 2c) as well as the unfractionated CM (Figure 2c). The lower MFI observed in 100 K vs the 10 K preparations is consistent with the reduced size of the EVs in 100 K preps observed by TEM and NTA (Figure 2a and b).

To assess the ability of flow cytometry to reveal size-based enrichment of EVs isolated by differential UC, EVs were mapped to sizing liposomes (90–400 nm) labeled with the same di8-staining Method #2 (Figure S3a and b). Given that PC3 cells have been previously characterized to shed L-EVs including Large Oncosomes (LO, ≥1 μm),^61,62^ we used the MFI of the largest sizing liposome (400 nm) as a landmark to delineate S-EV (<400 nm) from L-EV (≥400 nm) populations across the UC preparations (10 K vs 100 K, Figure 2d,e). Plotting the MFI of sizing liposome standards over scatter plots of 10 K and 100 K preparations from fibrosarcoma (HT1080) and prostate cancer (PC3) cells illustrates that our single EV flow cytometry method could indeed detect EVs across a large size range (Figure 2d,e). Consistent with the NTA observations (Figure 2b), this method detected a higher frequency of smaller EVs (<400 nm) in both 10 K and 100 K UC preparations, but a distinct enrichment (blue line) of larger EVs (≥400 nm) was observed in 10 K UC preparations (Figure 2e). This enrichment is 2-fold higher for PC3 than HT1080 cells (Figure 2e), an observation consistent with previously published reports.^61^

### EV Fingerprinting

During flow cytometry, individual EVs are detected when the fluorescence from the membrane-bound dye is sufficient to trigger the detector. Larger particles contain more lipid, and the corresponding fluorescence increases (Figure S3a,b). Lipophilic dyes such as di8, di-4-ANEPPDHQ and F2N12S are minimally fluorescent in aqueous solutions, but become strongly fluorescent upon intercalating into the hydrophobic environment of the lipid bilayer.^43,45,63−67^ In addition, the peak emission of these dyes shifts to a lower wavelength in membranes with increased liquid order.^68−70^ This emission shift for di8 was readily visualized using a panel of 1,2-distearoyl-*sn*-glycero-3-phosphocholine (DSPC) synthetic liposomes of 100 nm in which membrane order was transitioned from disordered (Ld) to ordered (Lo) through the incorporation of increasing amounts of cholesterol (Figure S3c–g) while keeping their size constant (Tables S4 and S5). With increasing cholesterol, the peak emission of di8-stained liposomes shifted from 702 to 611 nm without significantly altering the total fluorescence intensity (Figure S3d and 3e, respectively).

The complex shift in di8’s emission spectrum can be represented in a simplified manner using the Generalized Polarization (GP)^21,22,71−73^ calculation using the two peak emission channels for Lo and Ld as reported by di8 (Figure S3f,g).

**Figure 3:**
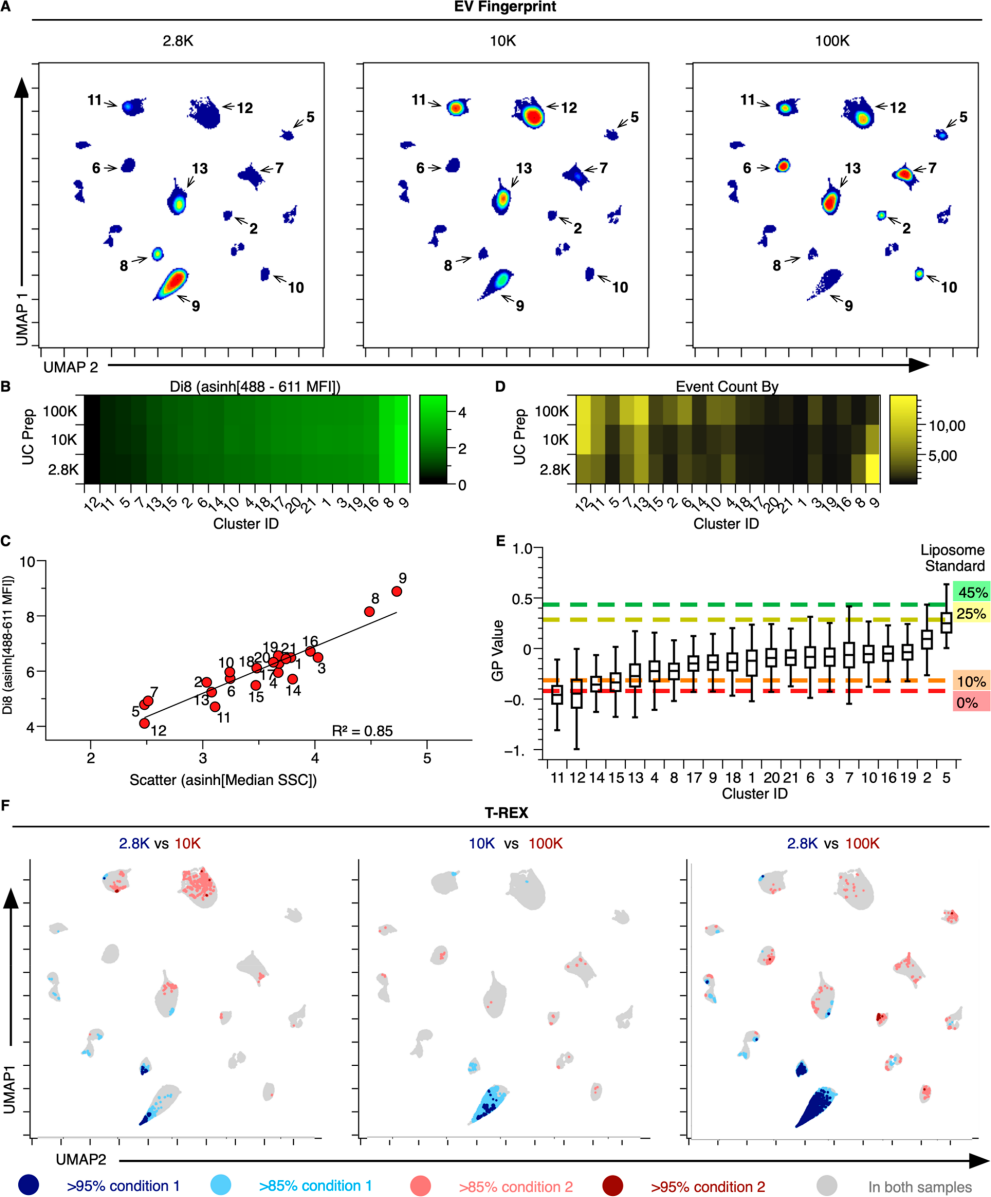
Enrichment of small and large EV populations corresponds to UC preparations. (A) EV Fingerprints of 2.8, 10, and 100 K DG-UC EV preparations generated sequentially from PC3 CM. Colors represent particle density from high (red) to low (blue). (B) Heatmap of clusters separated by DG-UC speed and sorted according to relative size (di8 signal, 488–611 median fluorescence intensity [MFI]) for each cluster. (C) Cluster plot of relative EV size mapping di8 fluorescence against scatter (488–611 MFI vs SCC, linear regression *R*^2^ = 0.85). (D) Heatmap of EV abundance per cluster, using the cluster ranking from panel B. (E) Box plot of the membrane order metric (GP value) for each cluster overlaid by a standard of membrane order composed of cholesterol-containing DSPC liposomes, which range from low membrane order (Ld, 0% cholesterol) to high membrane order (Lo, 45% cholesterol). (F) Independent pairwise evaluation of DG-UC EV preparations with T-REX Statistical thresholds of >85% and >95% expansion in the *k*-nearest neighbor (KNN) region denoted in red or blue, respectively. *k*-value = 60.

**Figure 4:**
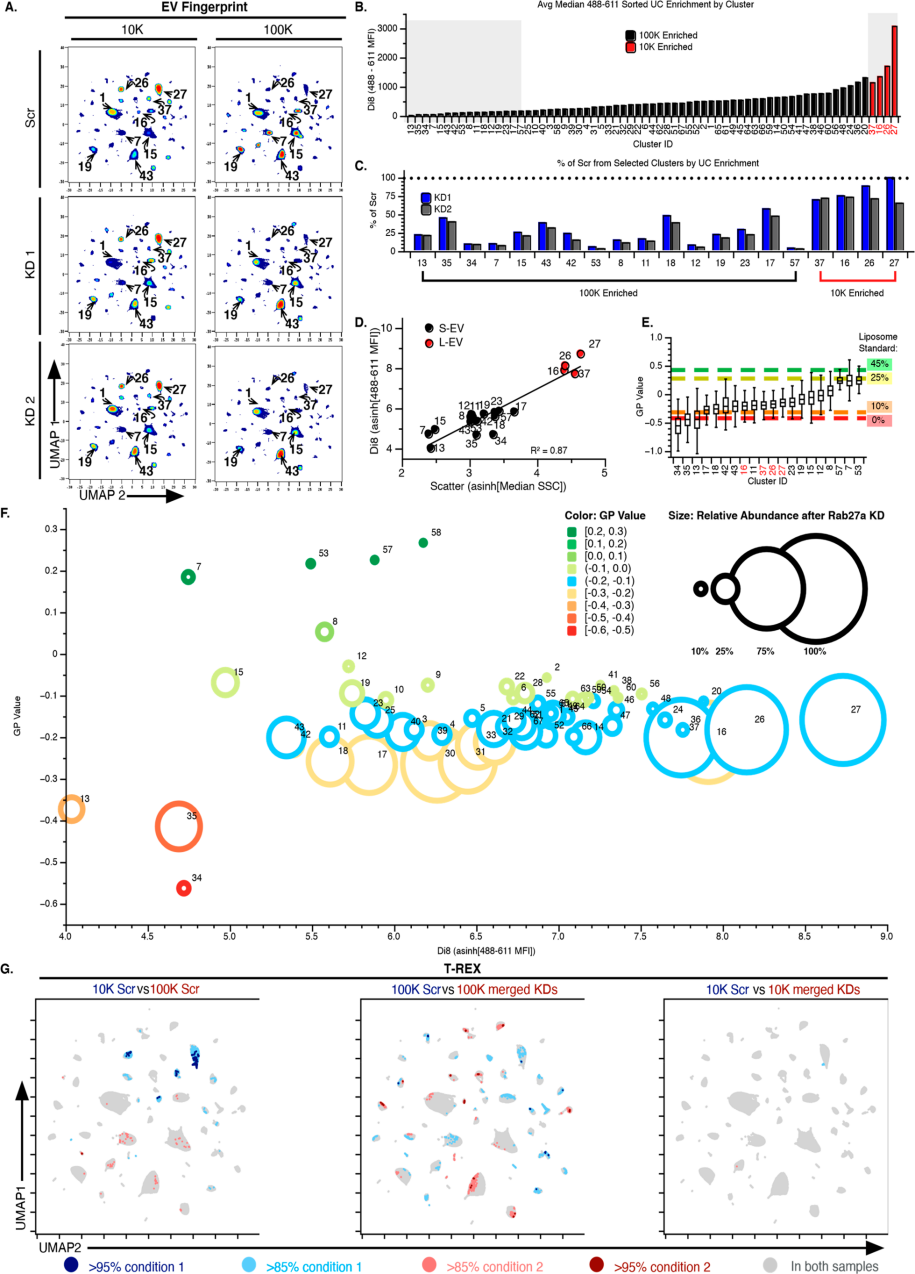
EV Fingerprinting resolves populations disrupted by EV secretion. (A) EV Fingerprints of 10 K (left) and 100 K (right) UC EV preparations from the control HT1080 cells (Scr, top panel) and the Rab27a KD HT1080 cells (KD1 and KD2 in the middle and bottom panel, respectively). Colors represent particle density from high (red) to low (blue). 80% sampling for a total of 1,102,755 events. (B) Clusters ranked by relative size (di8, 488–611 MFI). Black and red clusters correspond to S-EV and L-EV respectively, as defined in Figure S8e. Black = Clusters with ≥50% enriched in the 100 K. Red = Clusters >50% enriched in the 10 K. Gray boxes highlight clusters selected for further analysis in C–E. (C) Reduction of each EV population shown as a percentage of Scr for each cluster in KD1 (blue) and KD2 (gray) cells. (D) Cluster plot of relative EV size mapping di8 fluorescence against scatter (488–611 MFI vs SCC, linear regression: *R*^2^ = 0.87). Selected EV populations corresponding to S-EV and L-EV in “C” shown in black or red, respectively. (E) Box plot of the membrane order metric (GP value) for each EV population overlaid by a standard of membrane order composed of cholesterol-containing DSPC liposomes, which ranges from low membrane order (Ld, 0% cholesterol) to high membrane order (Lo, 45% cholesterol). (F) Visualization of the relative abundance for every EV population after Rab27a KD in the context of their relative size (*x*-axis) and membrane order (*y*-axis). (G) T-REX analysis on UMAP plots indicates regions of significant change when comparing: (i) EV preparations (10 KScr vs 100 KScr), (ii) Rab27a KD in S-EV preparations (100 K Scr vs 100 K merged KDs), and Rab27a KD in L-EV preparations (10 K Scr vs 10 K merged KDs, right). Direction and degree of change are shown in red and blue. *k*-value = 60.

**Figure 5:**
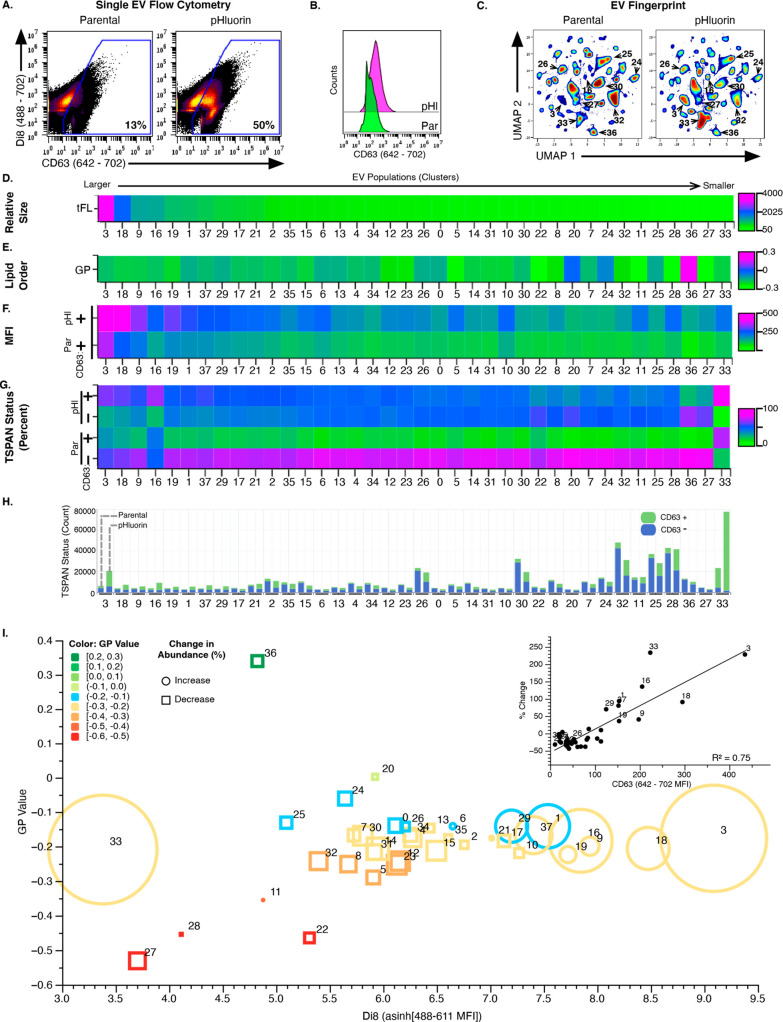
EV Fingerprinting identifies populations affected by CD63 overexpression (OE). (A) Scatter plot from HT1080 parental and pHluorin 100 K UC EV preparations showing gates for CD63 in parent and pHluorin EVs. (B) Histograms of CD63+ EVs gated antibody signal (642–702) from Parental and pHluorin 100 K EVs. (C) EV Fingerprints of HT1080 Parental and pHluorin 100 K EV preparations using di8 parameters (100% sampling for a total of 1,963,112 events). Colors represent particle densities from high (red) to low (blue). Cluster IDs shown represent the 10 most abundant populations. (D–G) Heatmaps of total fluorescence (tFL), membrane order (GP) and CD63 status by percent and count for EV populations ranked from large to small EVs (decreasing tFL). (D) Heatmap of total fluorescence (tFL). (E) Heatmap of membrane order (GP). (F) Heatmap of CD63 antibody signal (642–702 MFI) from Parental (Par) and pHluorin (pHl) EV populations. (G) Heatmap of CD63 status represented as the relative proportion of the population (%) for CD63– and CD63+ EVs from Parental (Par) and pHluorin (pHl) EVs. (H) Stacked bar plot with quantitative representation of CD63– (blue) and CD63+ (green) events in each population for Parental and pHluorin EVs. (I) Visualization of the relationship between relative size and membrane order of an EV population and the impact of CD63 overexpression. The relative abundance of each EV population after Rab27a KD was plotted against its di8 MFI and GP value. Inset illustrates the relationship of % change in abundance relative to CD63 (anti-CD63 MFI (642–702)) linear regression, *R*^2^ = 0.75.

The ability of EV flow cytometry to detect membrane order using di8 was validated by using two additional established reporters of membrane order (F2N12S^70,74−77^ and Di-4-ANEPPDHQ^70,78−80^) to distinguish between liposomes comprised of a highly ordered lipid bilayer (dipalmitoylphosphatidylcholine, DPPC) vs a disordered bilayer (1,2-dioleoyl-*sn*-glycero-3-phosphocholine, DOPC).^81,82^ All three dyes demonstrated a clear increase in the GP value when comparing liposomes of Lo and Ld bilayers (Figure 2f). A similar increase in GP value was observed when the membrane order of DOPC and DSPC/chol/DMG-PEG2000 liposomes were increased through the incorporation of cholesterol (Figure 2g and Figure S3h, respectively). GP values of di8 stained DOPC liposomes with increasing amounts of cholesterol were consistent across three different experimental sites, confirming the robustness of this readout (Figure S3i). Considering the superior stability, water solubility, and brightness of di8, only this dye was used in subsequent studies. Unlike synthetic liposomes, the lipid bilayer composition of biological membranes is complex and multiphasic.^34^ Since the emission spectrum of lipophilic dyes is influenced by the nature of their lipid environment, the complexity of an EV membrane can be captured by corresponding changes in the di8 fluorescence emission spectrum. To distinguish between EV populations with distinct spectral profiles, we leveraged this complexity through dimensional reduction of 20 features captured across 5 emission channels by the TDI-CCD camera of the CellStream (see Figure 1 for summary). Cluster analysis was subsequently used to deconvolve the heterogeneous EV populations detected by single EV flow cytometry into distinct populations. This analysis allowed us to determine which EV populations were differentially enriched during UC purification and which EV populations were impacted by molecular perturbation of EV biogenesis and secretion pathways (Figures 3, 4, and 5). We refer to this method of EV population analysis as “EV Fingerprinting”.

Four different dimensional reduction methods were considered for EV Fingerprinting, including Principal Component Analysis (PCA),^83^ t-distributed Stochastic Neighbor Embedding (t-SNE),^84^ Pairwise Controlled Manifold Approximation (PaCMAP),^85^ and UMAP.^51,52^ Clustering by HDBSCAN was used to assess the ability of each method to discern discrete populations (Figure S4a–c). The linear dimensional reduction method PCA failed to achieve reasonable separation while UMAP exhibited the best performance of the three nonlinear methods as evidenced by having the lowest number of unclassified events and the most well-defined clusters (Figure S4b,c). While UMAP was utilized in all subsequent analyses, all three algorithms identify clusters across a similar GP range, which further supports the conclusion that features derived from membrane order are driving the distinction between EV populations (Figure S4c).

An analysis workflow was constructed in the data science platform KNIME to facilitate the processing of flow cytometry files (.fcs) and perform EV Fingerprinting (Figure S5a–c).^86,87^ In brief, selected flow cytometry files are ingested after which the 20 EV features (Table S2 and Table S6) were transformed (asinh) and subsequently reduced to two dimensions using UMAP followed by clustering using HDBSCAN. The KNIME workflow not only facilitates data processing but also enables visualization and export of the data as tabular data or images.

To determine if EV Fingerprinting could be used to quantitatively assess heterogeneous particle populations, the method was used to analyze fluorescent sizing beads of known concentration and compared with manual gating (Figure S6). Indeed the analysis generated bead-specific clusters and distinguished them from background particles (Figure S6a,b). Quantitation was accurate for both manual and HDBSCAN gating as they detected the expected 1900 beads/μL at 1:4 dilution (dotted line, Figure S6c).

EV Fingerprinting was subsequently applied to EVs labeled intrinsically with fluorescently tagged CD63 EV-reporter (pHluorin-CD63)^50^ or extrinsically with di8. Flow cytometry of unpurified CM and purified EVs isolated by sequential 10 K and 100 K UC were used to evaluate the ability of EV Fingerprinting to assess the enrichment of EVs by ultracentrifugation. The quantitative range for EV flow cytometry was determined by serial dilution, as shown in Figure S2d. Intrinsically (pHluorin-CD63) and extrinsically (di8) labeled EV samples were analyzed by using the dimensional reduction workflow (Figure S7). It was possible to observe EV populations differentially enriched between 10 K and 100 K UC preparations in both intrinsically and extrinsically labeled EVs (10 K vs 100 K, Figure S7). However, the dimensional reduction of di8-labeled EVs revealed many more distinct EV populations than pHluorin-CD63 EVs (Figure S7, detection of pHluorin-CD63 vs detection of di8). Consequently, subsequent studies leveraged only extrinsic di8 labeling for EV Fingerprinting.

### EV Fingerprinting Captures Size-Based Enrichment of EV Populations by Density Gradient UC Purified EV

EVs are commonly isolated by sequential UC at incrementally increasing speeds (2,800*g*, 10,000*g*, and 100,000*g*) followed by centrifugal upward flotation in a density gradient (DG-UC).^60,88^ This method leverages the size and density of EVs to separate them from nonvesicular particles and proteins. Moreover, L-EVs are separated from S-EVs using slow speed UC (2,800–10,000*g*) while S-EVs are enriched in subsequent high-speed UC (100,000*g*).^62^

We tested the capacity of EV Fingerprinting to detect the differential enrichment of EV populations by DG-UC at 2,800*g* (2.8 K), 10,000*g* (10 K), and 100,000*g* (100 K) from PC3 cells (Figure 3). Tunable resistive pulse sensing (TRPS) sizing measurements of the isolated EV preparations from PC3 cells, known to secrete both L-EVs and S-EVs,^62^ confirmed larger vesicles (>200 nm) enriched in the 2.8 K and 10 K preparations compared to the 100 K preparation (Figure S8a–c). Flow cytometry of di8-labeled EV preparations readily detected EVs in all three preparations when compared to the buffer alone (Figure S8d). The detection of a low number of particles with high di8 MFI (i.e., larger EVs) in the 2.8 K preparation compared to a high number of particles with lower di8 MFI (i.e., smaller EVs) in the 100 K preparation is consistent with the TRPS measurements. By comparison, the 10 K preparation seems to contain a mixture of both larger and smaller EVs, as assessed by di8 MFI in flow cytometry and TRPS sizing. EV Fingerprinting of DG-UC purified EVs reveals over 80 distinct populations (Figure S8e). After removal of buffer-derived clusters and rank-ordering the populations according to abundance (Figure S8f), clusters containing ≥1% of total data were examined for differential enrichment between 2.8 K, 10 K and 100 K preparations (Figure 3).

The UMAP embedding reveals PC3 EV populations as clearly resolved clusters with visible sample-specific changes in particle abundance (Figure 3a). A heatmap of the clusters ranked according to di8 labeling (asinh[488–611 MFI]) suggests that the EV populations vary greatly in size, with cluster 9, enriched in the 2.8 K EV preparation, representing the brightest and therefore the largest EV population (comparing left to right; Figure 3b). This is confirmed when plotting the di8 fluorescence (MFI) of the population against its scatter (Figure 3c, linear regression *R*^2^ = 0.85). Importantly, each of the three preparations contains detectable quantities of every EV population with a similar MFI (Figure 3b). As expected, the relative enrichment of each EV population corresponds to their relative size (Figure 3d). Larger EVs (high MFI, i.e., clusters 8 and 9) are enriched in 2.8 K preparations while smaller EVs (low MFI, i.e., clusters 5, 7, 11, 12 and 13) are enriched in 100 K while the 10 K preparation contains a mixture of sizes (Figure 3d). These observations are consistent with TRPS measurements (Figure S8a) and published findings.^44^

The resolution of several EV populations with very similar di8 MFI (e.g., 5, 7, 11, and 12, Figure 3c) indicates that the dimensional reduction of EV Fingerprinting deconvolves EVs using multiple parameters. Indeed, when ranked according to their GP-value and mapped against cholesterol-containing membrane order standards, EV populations of similar size exhibit very different membrane order, with populations 11 and 13 exhibiting disordered membranes (low GP value) relative to populations 7 and 5 (high GP value) (Figure 3e). Interestingly, while S-EVs, enriched in 100 K preparations, range widely in their membrane order (GP −0.5 to +0.4), L-EVs enriched in the 2.8 K preparation (clusters 8 and 9) seem to be positioned at the midpoint of this range (GP −0.2 to +0.0, Figure 3e).

To ensure that the observed differential enrichment of EV populations was unbiased, we completed an unsupervised assessment with “Tracking Responders EXpanding” (T-REX) algorithm, which detects regions of significant change within phenotypically homogeneous events in a pairwise comparison of two conditions, such as low- or high-speed centrifugation.^89^ Using the same UMAP embeddings, three pairwise sample comparisons across 2.8 K, 10 K, and 100 K DG-UC with T-REX confirmed the size-based EV enrichment identified by analysis of individual HDBSCAN clusters (Figure 3f). The L-EV clusters 8 and 9 are identified by T-REX as enriched (≥85%) in the low-speed preparations while several S-EV clusters (including 5, 11, 12, and 13) were identified as enriched in the high-speed preparations. Thus, using EV Fingerprinting as a basis for differentiating between the EV populations, both conventional cluster analysis and T-REX identify populations of S-EVs and L-EVs that are enriched differentially by DG-UC.

### EV Fingerprinting Reveals That the Loss of Rab27a Differentially Impacts Select Small EV Populations

EV secretion through late endosomal/lysosomal compartments relies in many cells on a small GTPase, Rab27a, which controls MVB docking to the plasma membrane.^11^ Thus, the knockdown (KD) of Rab27a can reduce the secretion of exosomes through exocytosis.^8^ However, whether Rab27a regulates the secretion of most or distinct types of S-EVs is unclear. To address this question, we performed a comparative EV Fingerprinting analysis on 10 K and 100 K EVs purified from previously characterized Rab27a shRNA-mediated knockdowns (KD1 and KD2) and a scrambled shRNA control (Scr) in HT1080 cells and examined the impact on individual EV populations.^8,50^

The previously published reduction of S-EV secretion upon knockdown of Rab27a was confirmed by NTA analysis of both 10 K and 100 K UC EV preparations, with ∼50% reduction in EV numbers observed in the 100 K preparations and no reduction in EV numbers in the 10 K preparations (Figure S8a,b).^8,50^ The same 10 K and 100 K EV preparations from Rab27a KD and Scr were subsequently analyzed by flow cytometry after di8 labeling (Method #2). Congruent with NTA data, a reduced number of EVs were detected by EV flow cytometry for Rab27a KDs in the 100 K, but not the 10 K preparations (Figure S8c and d). EV preparations were further analyzed using the EV Fingerprinting workflow. Similar to the PC3 analysis in Figure 3, EV Fingerprinting revealed differential enrichment of EV populations across the HT1080 10 K and 100 K EV preparations (Scr 10 K vs 100 K, Figure 4a). Among these, several 100 K EV populations from Rab27a KD cells appear visibly reduced in the 100 K, while 10 K EV populations appear unaffected (KD1 and KD2, clusters 7, 15, and 19 vs 26 and 27, respectively, Figure 4a).

A detailed analysis of S-EVs and L-EVs was subsequently performed to determine whether the loss of Rab27a impacted select EV populations. For this evaluation the S-EV and L-EV populations were defined according to their relative enrichment in 100 K vs 10 K preparation and ranked according to their 488–611 MFI (Figure S8e and Figure 4b). S-EVs were subsequently defined as 100 K enriched EV populations in the lower quartile of the MFI range (black, Figure 4b) while L-EVs were defined as the 10 K enriched EV (red, Figure 4b). Since only four populations matched the L-EV criteria, all were included in the subsequent analyses.

The impact of Rab27a on select individual S-EV and L-EV populations (gray boxes, Figure 4b) was assessed. While the L-EVs are minimally impacted, the overall reduction in the S-EVs is readily apparent (Figure 4c). However, not all S-EV populations are impacted equally. Some EV populations were reduced to ≤10% of the levels found in the control Scr cell line (i.e., 7, 53, 12, and 57, Figure 4c) while others retained ≥40% of the original levels (i.e., 35, 43, 18, and 17, Figure 4c). This variation suggests that Rab27a has a selective impact on distinct S-EV populations.

This selective impact was investigated further by exploring the correlation of EV size (Figure 4d) and membrane order (Figure 4e) to Rab27a-dependent EV production. Analysis of the relative relationship between scatter and di8 fluorescence by plotting the di8 488–611 MFI against SSC validates the size stratification of S-EV and L-EV based on MFI (Figure 4d, *R*^2^ = 0.87). When ranked according to their GP value and mapped against cholesterol-containing standards it is evident that, similar to the PC3 S-EVs, the HT1080 S-EV populations vary greatly in their membrane order while the L-EV populations exhibit a midrange membrane order (Figure 4e vs Figure 3e).

To visualize the relationship between the relative size and membrane order of an EV population and its reliance on Rab27a for biogenesis, the relative abundance of each EV population after Rab27a KD was plotted against its di8 MFI and GP values (Figure 4f). It is immediately evident that S-EVs with high membrane order (green circles) are reduced to a much greater extent than S-EVs with a lower membrane order (blue, yellow, and red circles) by Rab27a KD (size of circles on graph indicates relative abundance of population after Rab27a KD). Indeed, regardless of EV size (as estimated by MFI), S-EV populations with a GP value greater than −0.15 are generally diminished more than S-EV populations with a lower membrane order (blue to red circles in Figure 4f).

Using T-REX^89^ on the same UMAP embeddings, three pairwise comparisons were performed as an unbiased confirmation of the effects of Rab27a KD on S-EVs (Figure 4g). The pairwise comparison for L-EV preparations (10 K) from Scr vs Rab27a KD cells revealed no significant changes, while a comparison for S-EV preparations (100 K) identified numerous populations impacted by the loss of Rab27a (Figure 4g). These observations corroborate the specificity of Rab27a perturbation on S-EVs while leaving the production of L-EVs mostly unaffected.

### EV Fingerprinting Identifies EV Populations Impacted by CD63 Overexpression

The TSPAN CD63 is a common EV cargo that has been shown to regulate biogenesis of S-EVs, but whose role is poorly understood.^62,90,91^ To gain an understanding of its role in generating EV heterogeneity, we evaluated the impact of CD63 expression on vesicle biogenesis by comparing the EV Fingerprint of HT1080 cells overexpressing CD63 (pHluorin-CD63) with that of control cells (HT1080-Parental). Consistent with Sung et al.,^50^ we observed an increase in EVs by NTA in the 100 K UC EV preparation from pHluorin-CD63 cells when compared to the 100 K UC EV preparation from parental cells (Parental vs pHluorin, Figure S10a). Elevated CD63 incorporation as an EV cargo from pHluorin-CD63 cells was confirmed by Western blotting (Figure S10b). Additionally, single-EV flow cytometry using dual labeling of di8 and anti-CD63 or control antibody (Iso, Method #2) confirmed the increase in CD63-positive (CD63+) EVs upon CD63 overexpression from 13% to 50% (Parental vs pHluorin, Figure 5a and Figure S10c). This is accompanied by an expected increase in CD63 fluorescence intensity (642–702) for pHluorin (pHl) EVs over Parental (Par) EVs (Figure 5b).

EV Fingerprinting of 100 K EV UC preparations from pHluorin-CD63 and Parental cells readily reveals increases and decreases in distinct EV populations upon CD63 overexpression (pHluorin vs Parental, Figure 5c). Heatmaps of these EV populations ordered by relative size from large to small using di8 MFI (total median fluorescence (tFL), Figure 5d) were evaluated for population-level changes of CD63 (Figure 5f–i).

As in Figure 3 and 4, the variations in membrane order are greater among the smaller EVs (GP Value, Figure 5e). Additionally, the amount of cargo per EV is more abundant in the larger EVs (CD63 MFI, Figure 5f). Expression of CD63-pHluorin increases this cargo in all EV populations (pHluorin versus Parental, Figure 5f). Indeed, both the % and absolute number of CD63 positive EVs are elevated (Figure 5g,h respectively). Interestingly, not all EV populations are impacted equally. The relative % of CD63+ EVs was nearly universally elevated (Figure 5g), but the number of CD63+ EVs did not increase equally: populations 33 and 3 exhibited a greater increase than all other populations. Indeed, a direct quantitative comparison of EV populations from Parental and pHluorin cells shows that populations 33 and 3 increased in both the absolute number of EVs and the number of CD63+ EVs, with population 33 having the largest gain in number (Figure 5h). Interestingly, the increase in the small EV population 33 was a very selective increase with concomitant decreases in the absolute number of EVs in other small EV populations (27, 36, 28, 25, 32, 24, 30 and others) despite the increased number (and relative proportion) of CD63+ EVs in those populations. Unexpectedly, there was also an increase in several of the larger EV populations (3, 18, 9, 16 and others). Unlike the impact of Rab27a KD (Figure 4), the expression of CD63 impacted EVs of intermediate liquid order and not Lo or Ld EVs (Figure 5i). In addition, the EV populations that are prominent recipients of the newly expressed CD63 as a percent (Figure 5g) were also the populations that increased in their relative abundance (Figure 5i, inset).

### Multiplex Detection of EV Cargo Reveals Divergent, Nonrandom Distribution Associated with EV Heterogeneity

**Figure 6:**
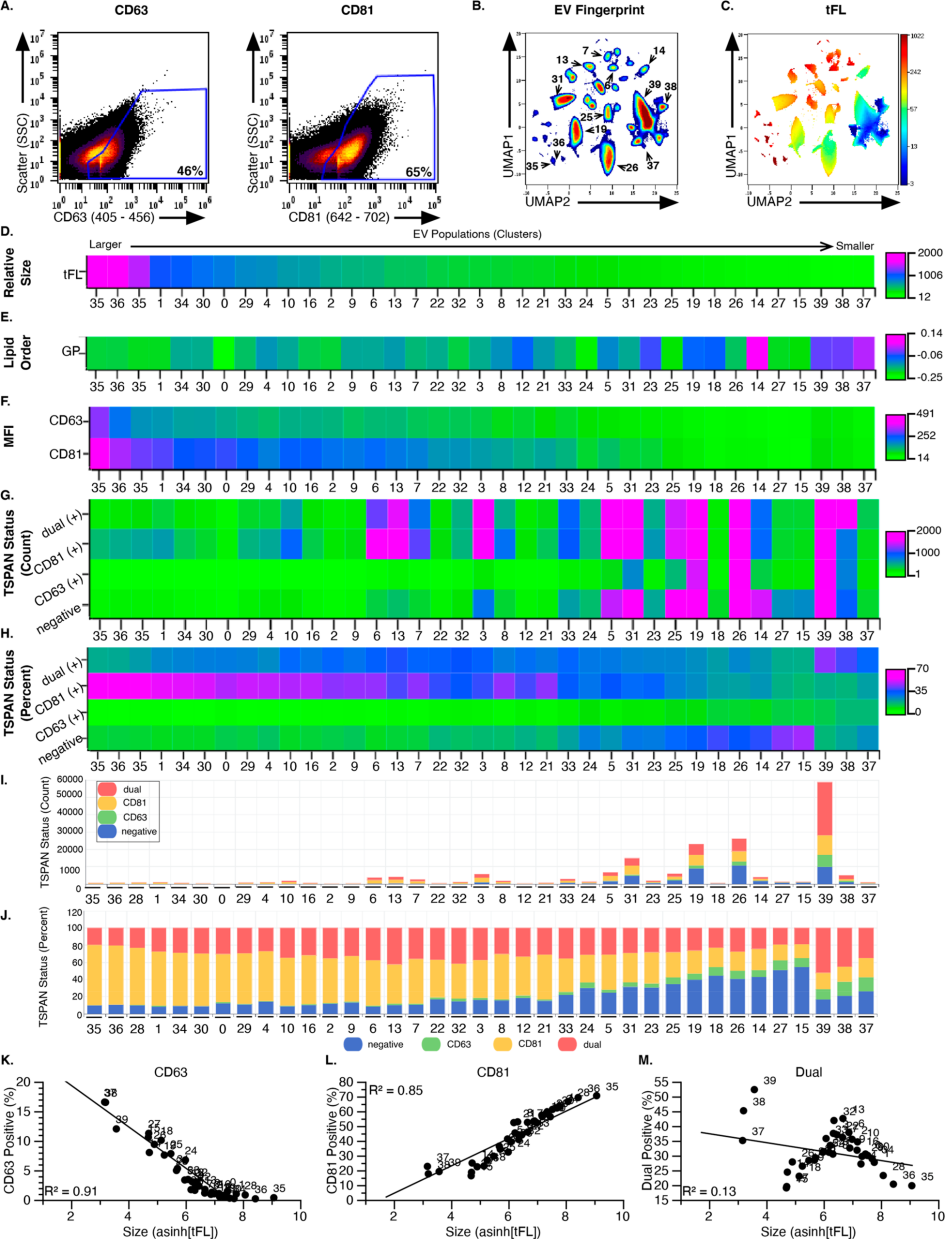
Multiplex analysis of the tetraspanins CD63 and CD81 using EV Fingerprinting reveals selective partitioning of cargos. (A) Scatter plots of 100 K HT1080 EV preparation after multiplex staining showing gates for CD63 and CD81 on the same sample. (B) EV Fingerprint of 100 K HT1080 EV after multiplex staining using only di8 parameters (100% of each sample was included for a total of 1,476,691 events). Colors represent particle density from high (red) to low (blue). (C) Total fluorescence (tFL) mapped onto the EV Fingerprint in B. Colors represent di8 MFI from high (red) to low (blue). (D–H) Heatmaps of total fluorescence (tFL), membrane order (GP), CD63 and CD81 intensity (MFI), and CD63 and CD81 status by percent and count for EV populations ranked from large to small EVs (decreasing tFL). (D) Heatmap of total fluorescence (tFL) and (E) Heatmap of lipid order (GP). (F) Heatmap of tetraspanin (TSPAN) antibody signal by cluster for CD81 (642–702) and CD63 (405–456). (G) Heatmap of TSPAN status represented by the number of events for TSPAN negative, single CD63+, single CD81+, and dual+ (CD63+ and CD81+) in each population. (H) Heatmap of TSPAN status represented as the relative proportion of the population (%). (I) Stacked bar plot with quantitative representation of negative (blue), single CD63+ (green), single CD81+ (yellow), and dual+ (red) events in each population. (J) Stacked bar plot representing the TSPAN status as a proportion (%) of each population. TSPAN negative (blue), single CD63+ (green), single CD81+ (yellow), and dual+ (red). (K–M) Linear regression analysis of TSPAN status vs EV size by population. CD63 positive (K, *R*^2^ = 0.91), CD81^+^ (L, *R*^2^ = 0.85), and dual positive (M, *R*^2^ = 0.13) assessment.

Given the selective impact of CD63 on EV biogenesis and its prevalence in small EVs, we leveraged multiplexing of two TSPAN cargos, CD63 and CD81, to determine if EV cargo may indeed have a nonrandom distribution in EV populations. To accomplish this, EV flow cytometry was performed on UC purified HT1080 S-EVs (100 K) after triple labeling with di8, and anti-CD63 and anti-CD81 antibodies (Method #2) followed by gating for EVs that were cargo negative, CD63-positive (CD63+), CD81-positive (CD81+), or positive for both TSPANs (dual+) (Figure 6a, Figure S11). EV Fingerprinting identified approximately 40 populations (Figure 6b) among which the variation in size can be visualized with a di8 MFI (tFL, Figure 6c). Heatmaps of these EV populations ordered by size from large to small were used to further evaluate population-level changes (Figure 6d–h). As in Figures 3–5, the variations in membrane order are greater among the smaller EVs, while the amount of cargo per EV is greater in the larger EVs (Figure 6d–f). As expected by our previous work showing that CD63 is an exosome marker,^8,50^ CD63+ EVs are more abundant in populations of smaller EVs, both as CD63+ and CD63/CD81 “dual+” EVs (Figure 6g–j). In contrast, CD81+ EVs are abundant in populations of both smaller and larger EVs (Figure 6g–j). Visualizing these cargo distributions quantitatively as stacked bar graphs underscores how CD63 and CD81 partition differently into EVs according to the size. This is particularly evident when plotting the cargo positive EVs as a percentage of the population.

We observed an increasing proportion of CD63 positive EVs in the small EVs, while the CD81 positive proportion increases in larger EVs (Figure 6j). Even though CD63 and CD81 are abundantly present in smaller EVs such as population 39, 26, 19, and 31 (Figure 6i), the proportion that contains CD81 increases for larger EVs while the proportion that contains CD63 increases for smaller EVs (Figure 6j). This observation is further validated by linear regression analysis of the % cargo positive EVs vs EV size (Figure 6k–m). The selective, nonrandom distribution of two distinct cargos into distinct EV populations further underscores the heterogeneity of EV populations.

## Discussion

EVs have been accredited with a wide variety of biological activities.^1^ The basis for these functions is thought to lie in the cargo composition of these vesicles which determines where and how they can impact biological processes.^5−8^ Indeed, bulk analysis of purified EV preparation and single-EV analysis have revealed that EVs vary greatly not only in size but also in cargo composition.^2^ To further investigate the heterogeneity of EVs, we developed “EV Fingerprinting” as a method of high-throughput, single-EV analysis that enables deconvolution of EV populations and their characterization from complex biological samples. Like other flow cytometry methods, EV Fingerprinting distinguishes itself from bulk EV analysis methods by allowing for the analysis of individual EVs and their cargo. However, unlike other flow methods, EV Fingerprinting leverages the dimensional reduction of 20 parameters collected from a fluorescent lipophilic environment-sensitive membrane probe that reports on both EV size and membrane order (Figure S3, S4 and Figure 2). The spectral shift in response to membrane order enables di8 to resolve far more EV heterogeneity than a static fluorophore, such as pHluorin-CD63 (Figure S7). Clustering the data based on these features revealed that cells produce a distinct array of EV populations characterized by the size and composition of their lipid membrane. The stability of these features is underscored by their ability to guide cluster formation for three distinct dimensional reduction algorithms (Figure S4). The final output of EV Fingerprinting is a quantifiable stratification of EV populations that enables the discovery of population-specific changes in response to molecular perturbation.

The ability of EV Fingerprinting to report accurately on changes in EV production was validated using orthogonal methods such as conventional flow cytometry, nanoparticle tracking, tunable resistive pulse sensing, and electron microscopy during the analysis of EV isolation using well established DG-UC. Similarly, the evaluation of cargo was validated by immunoblotting. However, unlike any established methods, EV Fingerprinting provided resolution of individual EV populations and enabled their characterization based on relative size, membrane order, and cargo composition.

Using EV Fingerprinting, we obtained evidence that supports the theory that EV heterogeneity is not stochastic but rather is controlled by underlying mechanisms of EV biogenesis. Specifically, molecular perturbations of EV production through loss of Rab27a or overexpression of CD63 were shown to impact select EV populations rather than perturb global EV production (Figure 4 and 5). Indeed, the loss of Rab27a selectively decreased the biogenesis of S-EV populations with high membrane order (Lo, Figure 4) while leaving L-EVs and S-EVs with low membrane order (Ld) relatively unaffected. Conversely, overexpressing EV cargo CD63 increased the abundance of EVs in one S-EV population while decreasing the abundance in several other S-EV populations. While both Rab27a and CD63 affect S-EV populations, especially exosomes,^8,50,92,93^ the role of Rab27a as an endolysosomal docking mediator is consistent with its ability to control a broader group of S-EVs. To further explore the possibility that cargo contributes to EV heterogeneity, we leveraged multiplexing of two distinct EV cargos to determine if they copartition equally into the same EVs. CD63 and CD81 are both transmembrane proteins of the TSPAN superfamily and established EV cargo.^17^ While CD63 is a well-established endosomal protein, CD81 is predominantly a cell-surface protein, suggesting that they are regulated by different trafficking processes.^94−99^ Multiplexing di8 with anti-CD63 and anti-CD81 antibodies revealed that, while the two TSPANs colocalize into a subset of the smallest S-EV populations, as individual cargo they exhibit opposing distributions across EV populations that correlates to size. Specifically, CD63 is preferentially distributed to smaller EVs while CD81 is preferentially distributed to larger EVs (Figure 6). These data may reflect to some extent their respective presence on endosomes and the plasma membrane.^90,100^ However, our finding that CD63 overexpression selectively boosts the number of EVs in one S-EV population despite leading to an overall increase in the percent of CD63-positive EVs across all populations (Figure 5) indicates a selective role for CD63 in EV biogenesis. This observation is a distinctive example of selective cargo partitioning contributing to vesicle heterogeneity in a nonrandom process that appears to be regulated by protein trafficking.

### Study Limitations

EV Fingerprinting is a multidimensional strategy to deconvolve the complexity of EV heterogeneity. However, as with all newly developed methods, EV Fingerprinting has limitations and potential for evolution. These include: (1) The data acquisition by flow cytometry relies on fluorescence triggering and is therefore limited by the instrument’s ability to discern the particles from background. Diluting the sample into background-free buffer or isolating the EVs may be needed for optimal detection of S-EVs. (2) Non-EV particles are commonly present in buffers and biological samples which complicates the detection of true EVs by the flow cytometer. In some instances, accurate detection requires the isolation of EVs through selective methods such as DG-UC. (3) EV Fingerprinting demonstrates the value of fluorescent, single-EV analysis. While a lipid incorporating dyes such as di8 should detect the entire EV population, antibody- or molecular marker- based detection (e.g., anti-CD63 or pHluorin-CD63) will only detect subsets of EVs. Indeed, EV analysis based only on molecular detection may be misleading with respect to the EV populations actually present. Consequently, new staining strategies will require corresponding calibration strategies to enable accurate interpretation. This study used liposomes of known lipid composition to validate membrane order in our flow cytometry-based assay, but new benchmarks will need to be established for future methods. (4) While the current method reveals the existence of distinct EV populations and enables their characterization, the function and the molecular makeup of these individual populations remains to be discovered. The current study was limited to antibody multiplexing, but applying our general method to sorting flow cytometry will allow for more detailed characterization with label-free methods such as mass spectrometry. In addition, performing gain-loss of function assays after molecular perturbation with fully characterized EV populations will elucidate which EV populations mediate specific biological activities.

## Conclusions

We introduce EV Fingerprinting as a single-EV analysis method. The ability of EV Fingerprinting to resolve distinct EV populations permits a detailed investigation of molecular processes regulating the biogenesis, composition, and biological function of EVs. In the future, this application may be extended to clinical specimens to aid in translational research, as well as biomarker development. Finally, results from analyses presented here suggest that heterogeneity in EV populations is not stochastic but rather is the product of specific regulation of biogenesis and protein trafficking.

## Methods

### Cell Culture and Reagents

HT1080 fibrosarcoma cells were maintained in Dulbecco’s Modified Eagle Medium (DMEM) high glucose (10-013-CV, Corning) supplemented with 10% bovine growth serum (BGS, SH30541.03, HyClone). HT1080 cells carrying the scrambled control or Rab27a-specific shRNAs (KD1 and KD2)^8^ or expressing pHluorin_M153R-CD63^50^ were cultured under the same conditions as the parental line. The prostate cancer cell line PC3 was obtained from the American Type Culture Collection (ATCC) and cultured in DMEM (Invitrogen). The DMEM was supplemented with 10% fetal bovine serum (Denville Scientific), 2 mM l-glutamine (Invitrogen), and 1% PenStrep (Invitrogen). The cells were grown at 37 °C and 5% CO_2_. Cell viability of the EV-producer cells was tested with the 0.4% Trypan Blue (Sigma) exclusion method. All cell lines were routinely tested for mycoplasma contamination using the MycoAlert PLUS Mycoplasma Detection Kit (Lonza).

Antibodies utilized included: anti-Rab27a (69298, Cell Signaling, 1:1,000 for WB), anti-β-actin (Ac-74, Sigma-Aldrich, 1:5,000 for WB), anti-CD63 (ab68418, Abcam, 1:500 for WB), and anti-GM130 (610822, BD Biosciences, 1:250 for WB). Horseradish peroxidase (HRP)-conjugated goat antimouse IgG (W4021, 1:10,000 for WB) or goat-antirabbit IgG (W4011, 1:10,000 for WB) were purchased from Promega. APC antihuman CD63 (353008, 1 μg/mL for FC), APC antihuman CD81 (349510, 1 μg/mL for FC), APC mouse IgG1, k isotype control (400120, 1 μg/mL for FC), BV421 antihuman CD63 (353030, 1 μg/mL for FC), and BV421 mouse IgG1, k isotype control (400158, 1 μg/mL for FC) antibodies were purchased from Biolegend.

### Extracellular Vesicle Isolation from Cultured Cells

Differential ultracentrifugation (UC) isolation was performed on HT1080-derived EV preparations as previously reported.^8,50^ Cell culture media was collected from cultures maintained at 80% confluent cells for 48 h in Opti-MEM (31985070, Thermo Fisher Scientific). The conditioned media were centrifuged at 300*g* for 5 min and 2,000*g* for 20 min to sediment live cells and cellular debris, respectively. The supernatant was centrifuged at 10,000*g* for 30 min (Ti45 rotor, Beckman Coulter) to collect L-EVs/10 K pellets. The supernatant from 10,000*g* centrifugation was further centrifuged at 100,000*g* for 18 h (Ti45 rotor) to pellet S-EVs/100 K pellets. Both the 10 K and 100 K pellets were resuspended in phosphate-buffered saline (PBS) and respectively spun again at 10,000*g* for 30 min or 100,000*g* for 3–6 h.

The isolation and density gradient purification of PC3 EVs was performed as reported with minor modifications described below.^12,101−103^ PC3 cells were grown in 18 × 150 mm cell culture dishes (Corning) until 90% confluence, washed in PBS and serum-starved for 24 h before the collection of cell conditioned media in serum free-medium (same DMEM as cell culture conditions, minus serum). The conditioned media was centrifuged at 300*g* × 3, 5 min each, to pellet down floating cells, followed by centrifugation at 2,800*g* for 10 min to pellet 2.8 K L-EVs. The resulting conditioned media was spun in an ultracentrifuge at 10,000*g* for 30 min (*k*-factor 2547.2) for the collection of 10 K L-EVs and the supernatant was then spun at 100,000*g* for 60 min (*k*-factor 254.7) for the collection of 100 K S-EV. All differential centrifugation steps were performed at 4 °C. 2.8 K, 10 K, and 100 K pellets were either resuspended in 0.2 μm-filtered PBS and used as differential UC pellets or subjected to further purification by density gradient ultracentrifugation (DG-UC) on Optiprep (Sigma) density gradients. Specifically, freshly pelleted EVs were resuspended in 0.2 μm-filtered PBS and deposited at the bottom of an ultracentrifuge tube. Next, 30% (4.3 mL, 1.20 g/mL), 25% (3 mL, 1.15 g/mL), 15% (2.5 mL, 1.10 g/mL), and 5% (6 mL, 1.08 g/mL) iodixanol solutions were sequentially layered at decreasing density to form a discontinuous gradient. Separation was performed by ultracentrifugation at 100,000*g* for 3 h 50 min (4 °C, *k*-factor 254.7) and EV-enriched fractions collected either at 1.10–1.15 g/mL for large EV or 1.10 g/mL for small EVs.^12^ Purified EVs were then washed in PBS (100,000*g*, 60 min, 4 °C) and resuspended in 0.2 μm-filtered PBS. All ultracentrifugation spins were performed in a SW28 swinging rotor (Beckman Coulter).

### Extracellular Vesicle Characterization

HT1080-derived pellets were resuspended in PBS and used fresh for NTA using ZetaView (Particle Metrix), Western blotting, or flow cytometry using the Amnis CellStream (Luminex). EV-specific marker expressions were validated in previous studies from our group.^8,50^ PC3-derived pellets were resuspended in PBS and characterized fresh through TRPS using qNano (Izon) and subsequently frozen at −80 °C before flow cytometry analysis.

### Liposome Preparation

1,2-Distearoyl-*sn*-glycero-3-phosphocholine (DSPC), 1,2-dipalmitoylphosphatidylcholine (DPPC), and 1,2-dioleoyl-*sn*-glycero-3-phosphocholine (DOPC), and cholesterol and 1,2-dimyristoyl-rac- glycero-3-methoxypolyethylene glycol-2000 (DMG-PEG2000) were obtained commercially (Avanti Polar Lipids). Liposomes were prepared through the established extrusion method.^104^ Briefly, lipids were desiccated for 2 h and allowed to reach RT. The lipids, cholesterol, and DMG-PEG2000 were weighed at the indicated ratios (Tables S4 and S5) and dissolved in chloroform, evaporated using a nitrogen stream, and left under vacuum overnight to form a lipid film. The film was rehydrated with PBS (pH 7.4) at 65 °C for 2 h with vortexing and the vesicles were further processed with five freeze (liquid nitrogen) and thaw (65 °C water bath) cycles. The nanoparticles were extruded through stacked polycarbonate filters (400, 200, or 100 nm) at least 10 times using a mini-extruder (Avanti Polar lipids, cat# 610000). The size of liposomes was measured using dynamic light scattering (Malvern Zetasizer).

### EV Staining for Flow Cytometry

#### Molecular Crowding Buffer (MC) Preparation and Storage

A 6.5% w/v solution of dextran, *M*_r_ = ∼100,000 (Millipore Sigma, Cat#: 09184) was prepared in DPBS without calcium and magnesium (Corning, Cat#: 21-031-CM) to make 2× MC Buffer and filtered with a 0.2 μm PVDF bottle filter (Millipore Sigma, Cat#: S2GVU05RE) in a sterile hood. 50 mL aliquots were stored at 4 °C until use.

#### Lipophilic Dye Preparation and Storage

The dyes Di-8-ANEPPS (Biotium, catalog no. 61012), F2N12S (3-hydroxyflavone, ThermoFisher, catalog no. A35137) and Di-4-ANEPPDHQ (ThermoFisher, catalog no. D36802) were prepared as recommended by the vendor. Dye solutions were then filtered with a 0.2 μm regenerated cellulose syringe filter (Corning, catalog no. 431215) and individual aliquots were stored at −20°C. Working solutions were prepared in DMSO (Millipore Sigma, catalog no. D8418) to a concentration of 5 mM. Only the aliquot actively being used was stored short-term at 4 °C. This 5 mM stock was diluted fresh in DMSO before each experiment to make the 25 μM working stocks used for staining buffers. Unless stated otherwise, the final staining concentration was 2 μM.

#### Di-8-ANEPPS Staining Strategies

The staining strategy used was dependent on the approximate particle concentration of the sample. The methods are outlined below.

Method #1 (Figure S1a): This staining method was used for samples with a low particle concentration (e.g., conditioned media or dilute purified EV) and is not amenable to multiplexing with antibody stains. EV-containing samples and serial dilutions were prepared in MC buffer and combined with the staining buffer prior to analysis (See Protocol). The final staining volume for each sample was 100 μL (50 μL of sample + 50 μL of staining buffer), and final di8 staining concentration was 0.25 μM. First, the staining buffer was prepared by diluting 25 μM di8 working stock into a 2× MC buffer for a dye concentration of 2.5 μM (1:50 in 50 μL of 2× MC per sample). Staining buffer was spun at 16,100*g* (max speed) at RT for 15 min. In the meantime, sample dilutions were prepared in PBS for a final volume of 50 μL. Finally, 50 μL of spun 2× staining MC buffer was added to 50 μL of diluted sample for a final staining volume of 100 μL, and di8 concentration of 0.25 μM. Samples were stained at RT in the dark for 1 h, then directly distributed in a 96-well round-bottom plate (Fisher Scientific, Cat#12565500) for flow analysis.

Method #2 (Figure S1b): This staining method was developed for samples with a high particle concentration (i.e., Purified EV samples, most synthetically derived nanoparticles, and liposomes) and supports the use of antibody staining. In principle, staining buffer and sample dilutions were prepared separately and combined for staining as in Method #1, but also includes a poststain 1:200 dilution. The final staining volume for each sample was 25 μL (14.5 μL of staining buffer + 10.5 μL of diluted sample OR 14.5 μL of staining buffer + 8 μL of diluted sample + 2.5 μL of antibody), and the staining concentration of di8 was 2 μM. First, the staining buffer was prepared by diluting 25 μM di8 working stock into 2× MC buffer. If used, antibodies were diluted in PBS to specified volumes (see the single antibody and multiplex protocol). Staining buffer and diluted antibodies were spun at 16,100*g* (max speed) at RT for 15 min protected from light. In the meantime, sample dilutions were prepared in PBS. Diluted sample, spun staining buffer, and spun antibody (if used) were then combined for the final di8 concentration of 2 μM and each antibody concentration 1 μg/mL, vortexed briefly and quickly spun to make sure the full 25 μL staining volume was collected at the bottom of the microcentrifuge tube, and stained for 1 h (−antibodies) or 3 h (+antibodies) at 37 °C in the dark. Poststain dilution tubes were then prepared with 1 mL of 1:1 MC buffer/PBS solution. Once staining was complete, samples were diluted 1:200 using the prepared poststain dilution tubes. Samples were then distributed in a 96-well round-bottomed plate for flow analysis.

#### NP-40 EV Lysis

EVs were stained using Method #2 above. After staining 1 h at 37 °C (prior to poststain dilution of 200×), 5 μL of 3% NP-40 was added to the stained sample making a final lysis volume of 30 μL at 0.5% NP-40. Samples were lysed for 15 min at RT, vortexing every 5 min. Finally, poststain dilution of 200× proceeded as in staining Method #2.

#### Flow Cytometry Data Acquisition Using the Amnis CellStream

All data was collected on an Amnis CellStream (Cytek) equipped with four lasers (405, 488, 561, and 642 nm) and a 96-well plate autosampler. The instrument was calibrated and initialized before each run according to the manufacturer’s instructions. Acquisition settings were then configured as follows: Small Particle Mode = ON, Flow Rate = SLOW (3.66 μL/min), Thresholds = ZERO, Trigger Channels = NONE, FSC/SSC Laser Power = 1%, 488 nm Laser Power = 25%, and if antibodies were used, 642 Laser Power = 50% and 405 Laser Power = 50%. Stopping criteria was set to TIME and data was collected for specified durations by experiment. Compensation was not performed.

#### Data Analysis

Initial data review and quality control was performed in Cytobank.^105^ Samples were assessed for the event count and signal intensity across dilutions. Specific signal was delineated from the background by comparing di8 and antibody-stained samples with buffer, unstained EV, and isotype controls. Data were plotted upon the asinh transformation. Samples with event counts in the quantitative range (Figure S7) were selected for analysis in the EV Fingerprinting workflow and subsequent data output was uploaded to Cytobank for visualization (Figure S5).

#### EV Fingerprinting Analysis Workflow

The analysis workflow was executed in the KNIME Analytics Platform^86,87^ using Python 3.6 running on a Mac Pro (early 2008, with 14 GB 800 mHz DDR2 FB-DIMM memory connected to an external storage array (RAID5).

The workflow consists of two stages (Figure S5). In the first stage, a randomized portion of each. fcs file is processed for exploration. In the second stage, clusters of interest can be re-examined by selecting and filtering clusters from the full data set for comprehensive analysis. In each stage the flow data is asinh transformed and embedded in 2D (UMAP),^51,52^ followed by cluster identification (HDBSCAN).^53,54^ Both UMAP and HDBSCAN algorithms can be parametrized externally (Table S6). Re-examination was executed similarly, using XGBoost to filter EVs belonging to the clusters of interest from the original sample files. For experiments with antibody staining, the antibody emission parameters were included in the parameter selection in addition to the di8 parameters (Table S2). EV population classification results were exported as. csv files for downstream analysis.

#### EV Population Analysis

Individual data files for each analyzed sample as well as a table containing data grouped by Cluster_ID were written and exported as csv files. UMAP plots were graphed in Cytobank by uploading individual data files to Cytobank using the Cluster_ID’s as “automatic cluster gates”, a column recognized by Cytobank as designated clusters for gating.

#### Quantitative Analyses

Statistical analyses and other graphing were performed in Excel (version 16.60), Prism (version 9.2.0), R (version 4.1.3) and DataGraph (version 4.7.1). 488–611 MFI by cluster was calculated from the grouped data table by including all samples in the cluster, excluding buffer controls. We considered an EV positive for an antibody when the number of events were absent or low (<1% of total) compared to the negative controls (dual stained buffer + antibody, dual stained EV sample + antibody). Statistical assessment of one biological replicate (*n* = 1) are shown in main figures. Qualitative assessment of trends was performed on biological replicates (*n* ≥ 2) for each experimental condition. In total, at least three biological replicates were analyzed for each experiment (*n* ≥ 3). Technical replicates were included in all experiments as serial dilutions or repeated reads.

Generalized Polarization (GP) Value Calculation:
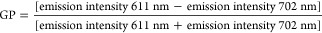


Total Fluorescence (tFL) Calculation:
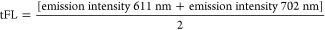


#### T-REX Analysis

An independent pairwise analysis of changes in EV populations was performed using a recently developed machine learning algorithm: T-REX (Tracking Responders EXpanding)^89^ in R (version 4.1.3). The data input for T-REX was a pair of samples in csv format generated by the EV Fingerprinting pipeline for each comparison of interest. In a comparison, T-REX equally sampled events from the paired data and used KNN with a *k*-value of 60 to find regions of difference between samples on the UMAP axes. These “hotspots” reflect both positive regions (elevation under condition one) and negative regions (elevation under condition two) across the pairwise sample comparisons. For KNN regions containing greater than or equal to 95% of events from one of the samples (dark blue for sample 1 and dark red for sample 2), events were clustered using DBSCAN (eps = 1, minPts = 1).^106^

### Degree of MISEV Compliance

Preanalytical variables such as isolation conditions^8,12,50,101−103^ and EV storage prior to analysis were reported as recommended by MISEV guidelines except for cell viability and number, which was not quantified but monitored visually.^47−49^ Size-based nomenclature was used unless morphology and protein cargo indicated specific cellular origination (e.g., Golgi). Protein markers used in the Western blot conform to MISEV recommendations. Limitations to optical imaging and other EV characterization methods were reported. Sample preparation and staining, including assay controls, were detailed in the Methods section. EV detection was assessed using serial dilutions to determine the quantitative range for every experiment and detergent-treated EV samples. The CellStream instrument was calibrated prior to every experiment by using vendor beads. Instruments were calibrated before each experiment, and settings for data acquisition were reported for every experiment. Given the limitations of the CellStream, conventional forward scatter for size calibration was not performed; instead, fluorescence vs SSC was used as a relative measure of EV size by cluster. EV characterization was performed using the EV Fingerprinting methodology; observations made by dimensional reduction of 20 parameters were further characterized by mapping defined EV populations against sizing and lipid composition standards.

## Data Availability

All codes related to EV Fingerprinting is accessible on KNIME HUB (https://hub.knime.com/-/spaces/-/~FkU8b4_sTscU1RlC/) KNIME HUB.

## Supporting Information Available

Figures S1–S11, and Tables S1–S6: Abbreviations list, list of metrics collected by CellStream; validation of quantitative particle detection using flow cytometry; optimized assay conditions for detecting EV by flow cytometry with Di-8-ANEPPS; summary of staining method conditions; validation of quantitative particle detection using flow cytometry; spectral emissions of Di-8-ANEPPS reflect particle size and membrane order; DSPC sizing liposome standards composition and size; DSPC cholesterol liposome standards composition and size; comparison of dimensional reduction approaches demonstrate optimal performance with UMAP; semiautomated analysis pipeline for dimensional reduction and clustering of EV populations; EV Fingerprinting pipeline clustering parameters; automated cluster-based gating is concordant with manual gating of synthetic sizing standards; sample selection criteria fitting for EV Fingerprinting; sizing characterization and di8 staining of DG-UC preps; Rab27a KD specifically affects 100 K EVs; Parental and pHluorin-CD63 100 K EV characterization and gating strategy; gating strategy for multiplex analysis of TSPANs (PDF)
Experiment-ready EV Fingerprinting protocols: Method 1 staining (XLSX)
Method 2 staining no antibody (XLSX)
Method 2 staining one antibody (XLSX)
Method 2 staining multiplex (XLSX)

## References

[ref1] Wortzel I.; Dror S.; Kenific C. M.; Lyden D. Exosome-Mediated Metastasis: Communication from a Distance. Dev Cell 2019, 49 (3), 347–360. 10.1016/j.devcel.2019.04.011. 31063754

[ref2] Van Niel G.; D’Angelo G.; Raposo G. Shedding Light on the Cell Biology of Extracellular Vesicles. Nat. Rev. Mol. Cell Biol. 2018, 19 (4), 213–228. 10.1038/nrm.2017.125. 29339798

[ref3] Colombo M.; Raposo G.; Théry C. Biogenesis, Secretion, and Intercellular Interactions of Exosomes and Other Extracellular Vesicles. Annu. Rev. Cell Dev Biol. 2014, 30, 255–289. 10.1146/annurev-cellbio-101512-122326. 25288114

[ref4] Zijlstra A.; Di Vizio D. Size Matters in Nanoscale Communication. Nat. Cell Biol. 2018, 20 (3), 228–230. 10.1038/s41556-018-0049-8. 29476154 PMC6652179

[ref5] van der Pol E.; Böing A. N.; Harrison P.; Sturk A.; Nieuwland R. Classification, Functions, and Clinical Relevance of Extracellular Vesicles. Pharmacol Rev. 2012, 64 (3), 676–705. 10.1124/pr.112.005983. 22722893

[ref6] Zhang Q.; Higginbotham J. N.; Jeppesen D. K.; Yang Y. P.; Li W.; McKinley E. T.; Graves-Deal R.; Ping J.; Britain C. M.; Dorsett K. A.; Hartman C. L.; Ford D. A.; Allen R. M.; Vickers K. C.; Liu Q.; Franklin J. L.; Bellis S. L.; Coffey R. J. Transfer of Functional Cargo in Exomeres. Cell Rep. 2019, 27 (3), 940–954. 10.1016/j.celrep.2019.01.009. 30956133 PMC6559347

[ref7] Sedgwick A. E.; Clancy J. W.; Olivia Balmert M.; D’Souza-Schorey C. Extracellular Microvesicles and Invadopodia Mediate Non-Overlapping Modes of Tumor Cell Invasion. Scientific Reports 2015 5:1 2015, 5 (1), 1–14. 10.1038/srep14748. PMC4602187 26458510

[ref8] Sung B. H.; Ketova T.; Hoshino D.; Zijlstra A.; Weaver A. M. Directional Cell Movement through Tissues Is Controlled by Exosome Secretion. Nat. Commun. 2015, 10.1038/ncomms8164. PMC4435734 25968605

[ref9] Tian F.; Zhang S.; Liu C.; Han Z.; Liu Y.; Deng J.; Li Y.; Wu X.; Cai L.; Qin L.; Chen Q.; Yuan Y.; Liu Y.; Cong Y.; Ding B.; Jiang Z.; Sun J. Protein Analysis of Extracellular Vesicles to Monitor and Predict Therapeutic Response in Metastatic Breast Cancer. Nat. Commun. 2021, 10.1038/s41467-021-22913-7. PMC8100127 33953198

[ref10] Zhou E.; Li Y.; Wu F.; Guo M.; Xu J.; Wang S.; Tan Q.; Ma P.; Song S.; Jin Y. Circulating Extracellular Vesicles Are Effective Biomarkers for Predicting Response to Cancer Therapy. eBioMedicine 2021, 67, 103365 10.1016/j.ebiom.2021.103365. 33971402 PMC8121992

[ref11] Ostrowski M.; Carmo N. B.; Krumeich S.; Fanget I.; Raposo G.; Savina A.; Moita C. F.; Schauer K.; Hume A. N.; Freitas R. P.; Goud B.; Benaroch P.; Hacohen N.; Fukuda M.; Desnos C.; Seabra M. C.; Darchen F.; Amigorena S.; Moita L. F.; Thery C. Rab27a and Rab27b Control Different Steps of the Exosome Secretion Pathway. Nat. Cell Biol. 2010, 12 (1), 19–30. 10.1038/ncb2000. 19966785

[ref12] Minciacchi V. R.; You S.; Spinelli C.; Morley S.; Zandian M.; Aspuria P. J.; Cavallini L.; Ciardiello C.; Sobreiro M. R.; Morello M.; Kharmate G.; Jang S. C.; Kim D. K.; Hosseini-Beheshti E.; Guns E. T.; Gleave M.; Gho Y. S.; Mathivanan S.; Yang W.; Freeman M. R.; Di Vizio D. Large Oncosomes Contain Distinct Protein Cargo and Represent a Separate Functional Class of Tumor-Derived Extracellular Vesicles. Oncotarget 2015, 6 (13), 11327–11341. 10.18632/oncotarget.3598. 25857301 PMC4484459

[ref13] Su H.; Rustam Y. H.; Masters C. L.; Makalic E.; McLean C. A.; Hill A. F.; Barnham K. J.; Reid G. E.; Vella L. J. Characterization of Brain-Derived Extracellular Vesicle Lipids in Alzheimer’s Disease. J. Extracell. Vesicles 2021, 10.1002/jev2.12089. PMC8111496 34012516

[ref14] Bonsergent E.; Grisard E.; Buchrieser J.; Schwartz O.; Théry C.; Lavieu G. Quantitative Characterization of Extracellular Vesicle Uptake and Content Delivery within Mammalian Cells. Nat. Commun. 2021, 10.1038/s41467-021-22126-y. PMC7994380 33767144

[ref15] Cai J.; Guan W.; Tan X.; Chen C.; Li L.; Wang N.; Zou X.; Zhou F.; Wang J.; Pei F.; Chen X.; Luo H.; Wang X.; He D.; Zhou L.; Jose P. A.; Zeng C. SRY Gene Transferred by Extracellular Vesicles Accelerates Atherosclerosis by Promotion of Leucocyte Adherence to Endothelial Cells. Clin Sci. (Lond) 2015, 129 (3), 259–269. 10.1042/CS20140826. 25783200

[ref16] Chen X.; Jia M.; Liu L.; Qiu X.; Zhang H.; Yu X.; Gu W.; Qing G.; Li Q.; Hu X.; Wang R.; Zhao X.; Zhang L.; Wang X.; Durkan C.; Wang N.; Wang G.; Luo Y. High-Fidelity Determination and Tracing of Small Extracellular Vesicle Cargoes. Small 2020, 10.1002/smll.202002800. 32877016

[ref17] Andreu Z.; Yáñez-Mó M. Tetraspanins in Extracellular Vesicle Formation and Function. Front. Immunol. 2014, 10.3389/fimmu.2014.00442. PMC4165315 25278937

[ref18] Haraszti R. A.; Didiot M. C.; Sapp E.; Leszyk J.; Shaffer S. A.; Rockwell H. E.; Gao F.; Narain N. R.; DiFiglia M.; Kiebish M. A.; Aronin N.; Khvorova A. High-Resolution Proteomic and Lipidomic Analysis of Exosomes and Microvesicles from Different Cell Sources. J. Extracell. Vesicles 2016, 10.3402/jev.v5.32570. PMC5116062 27863537

[ref19] Donoso-Quezada J.; Ayala-Mar S.; González-Valdez J. The Role of Lipids in Exosome Biology and Intercellular Communication: Function, Analytics and Applications. Traffic 2021, 22 (7), 204–220. 10.1111/tra.12803. 34053166 PMC8361711

[ref20] Semrau S.; Schmidt T. Membrane Heterogeneity - From Lipid Domains to Curvature Effects. Soft Matter 2009, 5 (17), 3174 10.1039/b901587f.

[ref21] Soto-Arriaza M. A.; Olivares-Ortega C.; Quina F. H.; Aguilar L. F.; Sotomayor C. P. Effect of Cholesterol Content on the Structural and Dynamic Membrane Properties of DMPC/DSPC Large Unilamellar Bilayers. Biochim. Biophys. Acta Biomembr 2013, 1828 (11), 2763 10.1016/j.bbamem.2013.07.031. 23954586

[ref22] Bagatolli L. A.; Gratton E. A Correlation between Lipid Domain Shape and Binary Phospholipid Mixture Composition in Free Standing Bilayers: A Two-Photon Fluorescence Microscopy Study. Biophys. J. 2000, 79 (1), 434–447. 10.1016/S0006-3495(00)76305-3. 10866969 PMC1300947

[ref23] Shimshick E. J.; Mcconnell H. M. Lateral Phase Separation in Phospholipid Membranes. Biochemistry 1973, 12 (12), 2351 10.1021/bi00736a026. 4351059

[ref24] Silvius J. R.; Del Giudice D.; Lafleur M. Cholesterol at Different Bilayer Concentrations Can Promote or Antagonize Lateral Segregation of Phospholipids of Differing Acyl Chain Length. Biochemistry 1996, 35 (48), 15198 10.1021/bi9615506. 8952467

[ref25] Kaiser H. J.; Lingwood D.; Levental I.; Sampaio J. L.; Kalvodova L.; Rajendran L.; Simons K. Order of Lipid Phases in Model and Plasma Membranes. Proc. Natl. Acad. Sci. U. S. A. 2009, 106 (39), 16645–16650. 10.1073/pnas.0908987106. 19805351 PMC2757813

[ref26] Cinek T.; Horejsí V. The Nature of Large Noncovalent Complexes Containing Glycosyl-Phosphatidylinositol-Anchored Membrane Glycoproteins and Protein Tyrosine Kinases. J. Immunol. 1992, 149 (7), 2262 10.4049/jimmunol.149.7.2262. 1382093

[ref27] Simons K.; Van Meer G. Lipid Sorting in Epithelial Cells. Biochemistry 1988, 27 (17), 6197 10.1021/bi00417a001. 3064805

[ref28] Brown D. A.; Rose J. K. Sorting of GPI-Anchored Proteins to Glycolipid-Enriched Membrane Subdomains during Transport to the Apical Cell Surface. Cell 1992, 68 (3), 533 10.1016/0092-8674(92)90189-J. 1531449

[ref29] Hønger T.; Jørgensen K.; Biltonen R. L.; Mouritsen O. G. Systematic Relationship between Phospholipase A2 Activity and Dynamic Lipid Bilayer Microheterogeneity. Biochemistry 1996, 35 (28), 9003 10.1021/bi960866a. 8703902

[ref30] Ursell T. S.; Klug W. S.; Phillips R. Morphology and Interaction between Lipid Domains. Proc. Natl. Acad. Sci. U. S. A. 2009, 106 (32), 13301 10.1073/pnas.0903825106. 19620730 PMC2726347

[ref31] Jørgensen K.; Mouritsen O. G. Phase Separation Dynamics and Lateral Organization of Two-Component Lipid Membranes. Biophys. J. 1995, 69 (3), 942 10.1016/S0006-3495(95)79968-4. 8519994 PMC1236323

[ref32] Corcoran J. A.; Salsman J.; De Antueno R.; Touhami A.; Jericho M. H.; Clancy E. K.; Duncan R. The P14 Fusion-Associated Small Transmembrane (FAST) Protein Effects Membrane Fusion from a Subset of Membrane Microdomains. J. Biol. Chem. 2006, 281 (42), 31778–31789. 10.1074/jbc.M602566200. 16936325

[ref33] Simons K.; Ikonen E. Functional Rafts in Cell Membranes. Nature 1997, 387 (6633), 569–572. 10.1038/42408. 9177342

[ref34] Skotland T.; Llorente A.; Sandvig K. Lipids in Extracellular Vesicles: What Can Be Learned about Membrane Structure and Function?. Cold Spring Harb. Perspect. Biol. 2023, 15 (8), a041415 10.1101/cshperspect.a041415. 37277192 PMC10411865

[ref35] Brzozowski J. S.; Jankowski H.; Bond D. R.; McCague S. B.; Munro B. R.; Predebon M. J.; Scarlett C. J.; Skelding K. A.; Weidenhofer J. Lipidomic Profiling of Extracellular Vesicles Derived from Prostate and Prostate Cancer Cell Lines. Lipids Health Dis. 2018, 10.1186/s12944-018-0854-x. PMC6128989 30193584

[ref36] Cohn W.; Melnik M.; Huang C.; Teter B.; Chandra S.; Zhu C.; McIntire L. B.; John V.; Gylys K. H.; Bilousova T. Multi-Omics Analysis of Microglial Extracellular Vesicles From Human Alzheimer’s Disease Brain Tissue Reveals Disease-Associated Signatures. Front. Pharmacol. 2021, 10.3389/fphar.2021.766082. PMC8675946 34925024

[ref37] Chandler W. L.; Yeung W.; Tait J. F. A New Microparticle Size Calibration Standard for Use in Measuring Smaller Microparticles Using a New Flow Cytometer. J. Thromb Haemost 2011, 9 (6), 1216–1224. 10.1111/j.1538-7836.2011.04283.x. 21481178

[ref38] Stoner S. A.; Duggan E.; Condello D.; Guerrero A.; Turk J. R.; Narayanan P. K.; Nolan J. P. High Sensitivity Flow Cytometry of Membrane Vesicles. Cytometry Part A 2016, 89 (2), 196–206. 10.1002/cyto.a.22787. 26484737

[ref39] Chandler W. L.; Yeung W.; Tait J. F. A New Microparticle Size Calibration Standard for Use in Measuring Smaller Microparticles Using a New Flow Cytometer. J. Thromb Haemost 2011, 9 (6), 1216–1224. 10.1111/j.1538-7836.2011.04283.x. 21481178

[ref40] Tian Y.; Gong M.; Hu Y.; Liu H.; Zhang W.; Zhang M.; Hu X.; Aubert D.; Zhu S.; Wu L.; Yan X. Quality and Efficiency Assessment of Six Extracellular Vesicle Isolation Methods by Nano-Flow Cytometry. J. Extracell. Vesicles 2020, 10.1080/20013078.2019.1697028. PMC6896440 31839906

[ref41] Lucchetti D.; Battaglia A.; Ricciardi-Tenore C.; Colella F.; Perelli L.; De Maria R.; Scambia G.; Sgambato A.; Fattorossi A. Measuring Extracellular Vesicles by Conventional Flow Cytometry: Dream or Reality. Int. J. Mol. Sci. 2020, 21 (17), 6257 10.3390/ijms21176257. 32872424 PMC7503575

[ref42] Welsh J. A.; Jones J. C.; Tang V. A. Fluorescence and Light Scatter Calibration Allow Comparisons of Small Particle Data in Standard Units across Different Flow Cytometry Platforms and Detector Settings. Cytometry A 2020, 97 (6), 592–601. 10.1002/cyto.a.24029. 32476280 PMC8482305

[ref43] Bondelli G.; Paternò G. M.; Lanzani G. Fluorescent Probes for Optical Investigation of the Plasma Membrane. Optical Materials: X 2021, 12, 100085 10.1016/j.omx.2021.100085.

[ref44] Bouquiaux C.; Castet F.; Champagne B. Unravelling the Effects of Cholesterol on the Second-Order Nonlinear Optical Responses of Di-8-ANEPPS Dye Embedded in Phosphatidylcholine Lipid Bilayers. J. Phys. Chem. B 2021, 125 (36), 10195–10212. 10.1021/acs.jpcb.1c05630. 34491062

[ref45] Jin L.; Millard A. C.; Wuskell J. P.; Dong X.; Wu D.; Clark H. A.; Loew L. M. Characterization and Application of a New Optical Probe for Membrane Lipid Domains. Biophys. J. 2006, 90 (7), 2563 10.1529/biophysj.105.072884. 16415047 PMC1403187

[ref46] Clarke R. J.; Lüpfert C. Influence of Anions and Cations on the Dipole Potential of Phosphatidylcholine Vesicles: A Basis for the Hofmeister Effect. Biophys. J. 1999, 76 (5), 2614–2624. 10.1016/S0006-3495(99)77414-X. 10233076 PMC1300231

[ref47] Erdbrügger U.; Lannigan J. Analytical Challenges of Extracellular Vesicle Detection: A Comparison of Different Techniques. Cytometry A 2016, 89 (2), 123–134. 10.1002/cyto.a.22795. 26651033

[ref48] Théry C.; Witwer K. W.; Aikawa E.; Alcaraz M. J.; Anderson J. D.; Andriantsitohaina R.; Antoniou A.; Arab T.; Archer F.; Atkin-Smith G. K.; Ayre D. C.; Bach J. M.; Bachurski D.; Baharvand H.; Balaj L.; Baldacchino S.; Bauer N. N.; Baxter A. A.; Bebawy M.; Beckham C.; Bedina Zavec A.; Benmoussa A.; Berardi A. C.; Bergese P.; Bielska E.; Blenkiron C.; Bobis-Wozowicz S.; Boilard E.; Boireau W.; Bongiovanni A.; Borràs F. E.; Bosch S.; Boulanger C. M.; Breakefield X.; Breglio A. M.; Brennan M.; Brigstock D. R.; Brisson A.; Broekman M. L. D.; Bromberg J. F.; Bryl-Górecka P.; Buch S.; Buck A. H.; Burger D.; Busatto S.; Buschmann D.; Bussolati B.; Buzás E. I.; Byrd J. B.; Camussi G.; Carter D. R. F.; Caruso S.; Chamley L. W.; Chang Y. T.; Chaudhuri A. D.; Chen C.; Chen S.; Cheng L.; Chin A. R.; Clayton A.; Clerici S. P.; Cocks A.; Cocucci E.; Coffey R. J.; Cordeiro-da-Silva A.; Couch Y.; Coumans F. A. W.; Coyle B.; Crescitelli R.; Criado M. F.; D’Souza-Schorey C.; Das S.; de Candia P.; De Santana E. F.; De Wever O.; del Portillo H. A.; Demaret T.; Deville S.; Devitt A.; Dhondt B.; Di Vizio D.; Dieterich L. C.; Dolo V.; Dominguez Rubio A. P.; Dominici M.; Dourado M. R.; Driedonks T. A. P.; Duarte F. V.; Duncan H. M.; Eichenberger R. M.; Ekström K.; EL Andaloussi S.; Elie-Caille C.; Erdbrügger U.; Falcón-Pérez J. M.; Fatima F.; Fish J. E.; Flores-Bellver M.; Försönits A.; Frelet-Barrand A.; Fricke F.; Fuhrmann G.; Gabrielsson S.; Gámez-Valero A.; Gardiner C.; Gärtner K.; Gaudin R.; Gho Y. S.; Giebel B.; Gilbert C.; Gimona M.; Giusti I.; Goberdhan D. C. I.; Görgens A.; Gorski S. M.; Greening D. W.; Gross J. C.; Gualerzi A.; Gupta G. N.; Gustafson D.; Handberg A.; Haraszti R. A.; Harrison P.; Hegyesi H.; Hendrix A.; Hill A. F.; Hochberg F. H.; Hoffmann K. F.; Holder B.; Holthofer H.; Hosseinkhani B.; Hu G.; Huang Y.; Huber V.; Hunt S.; Ibrahim A. G. E.; Ikezu T.; Inal J. M.; Isin M.; Ivanova A.; Jackson H. K.; Jacobsen S.; Jay S. M.; Jayachandran M.; Jenster G.; Jiang L.; Johnson S. M.; Jones J. C.; Jong A.; Jovanovic-Talisman T.; Jung S.; Kalluri R.; Kano S. i.; Kaur S.; Kawamura Y.; Keller E. T.; Khamari D.; Khomyakova E.; Khvorova A.; Kierulf P.; Kim K. P.; Kislinger T.; Klingeborn M.; Klinke D. J.; Kornek M.; Kosanović M. M.; Kovács Á. F.; Krämer-Albers E. M.; Krasemann S.; Krause M.; Kurochkin I. V.; Kusuma G. D.; Kuypers S.; Laitinen S.; Langevin S. M.; Languino L. R.; Lannigan J.; Lässer C.; Laurent L. C.; Lavieu G.; Lázaro-Ibáñez E.; Le Lay S.; Lee M. S.; Lee Y. X. F.; Lemos D. S.; Lenassi M.; Leszczynska A.; Li I. T. S.; Liao K.; Libregts S. F.; Ligeti E.; Lim R.; Lim S. K.; Line A.; Linnemannstöns K.; Llorente A.; Lombard C. A.; Lorenowicz M. J.; Lörincz Á. M.; Lötvall J.; Lovett J.; Lowry M. C.; Loyer X.; Lu Q.; Lukomska B.; Lunavat T. R.; Maas S. L. N.; Malhi H.; Marcilla A.; Mariani J.; Mariscal J.; Martens-Uzunova E. S.; Martin-Jaular L.; Martinez M. C.; Martins V. R.; Mathieu M.; Mathivanan S.; Maugeri M.; McGinnis L. K.; McVey M. J.; Meckes D. G.; Meehan K. L.; Mertens I.; Minciacchi V. R.; Möller A.; Møller Jørgensen M.; Morales-Kastresana A.; Morhayim J.; Mullier F.; Muraca M.; Musante L.; Mussack V.; Muth D. C.; Myburgh K. H.; Najrana T.; Nawaz M.; Nazarenko I.; Nejsum P.; Neri C.; Neri T.; Nieuwland R.; Nimrichter L.; Nolan J. P.; Nolte-’t Hoen E. N. M.; Noren Hooten N.; O’Driscoll L.; O’Grady T.; O’Loghlen A.; Ochiya T.; Olivier M.; Ortiz A.; Ortiz L. A.; Osteikoetxea X.; Ostegaard O.; Ostrowski M.; Park J.; Pegtel D. M.; Peinado H.; Perut F.; Pfaffl M. W.; Phinney D. G.; Pieters B. C. H.; Pink R. C.; Pisetsky D. S.; Pogge von Strandmann E.; Polakovicova I.; Poon I. K. H.; Powell B. H.; Prada I.; Pulliam L.; Quesenberry P.; Radeghieri A.; Raffai R. L.; Raimondo S.; Rak J.; Ramirez M. I.; Raposo G.; Rayyan M. S.; Regev-Rudzki N.; Ricklefs F. L.; Robbins P. D.; Roberts D. D.; Rodrigues S. C.; Rohde E.; Rome S.; Rouschop K. M. A.; Rughetti A.; Russell A. E.; Saá P.; Sahoo S.; Salas-Huenuleo E.; Sánchez C.; Saugstad J. A.; Saul M. J.; Schiffelers R. M.; Schneider R.; Schøyen T. H.; Scott A.; Shahaj E.; Sharma S.; Shatnyeva O.; Shekari F.; Shelke G. V.; Shetty A. K.; Shiba K.; Siljander P. R. M.; Silva A. M.; Skowronek A.; Snyder O. L.; Soares R. P.; Sódar B. W.; Soekmadji C.; Sotillo J.; Stahl P. D.; Stoorvogel W.; Stott S. L.; Strasser E. F.; Swift S.; Tahara H.; Tewari M.; Timms K.; Tiwari S.; Tixeira R.; Tkach M.; Toh W. S.; Tomasini R.; Torrecilhas A. C.; Tosar J. P.; Toxavidis V.; Urbanelli L.; Vader P.; van Balkom B. W. M.; van der Grein S. G.; Van Deun J.; van Herwijnen M. J. C.; Van Keuren-Jensen K.; van Niel G.; van Royen M. E.; van Wijnen A. J.; Vasconcelos M. H.; Vechetti I. J.; Veit T. D.; Vella L. J.; Velot É.; Verweij F. J.; Vestad B.; Viñas J. L.; Visnovitz T.; Vukman K. V.; Wahlgren J.; Watson D. C.; Wauben M. H. M.; Weaver A.; Webber J. P.; Weber V.; Wehman A. M.; Weiss D. J.; Welsh J. A.; Wendt S.; Wheelock A. M.; Wiener Z.; Witte L.; Wolfram J.; Xagorari A.; Xander P.; Xu J.; Yan X.; Yáñez-Mó M.; Yin H.; Yuana Y.; Zappulli V.; Zarubova J.; Žėkas V.; Zhang J.; Zhao Z.; Zheng L.; Zheutlin A. R.; Zickler A. M.; Zimmermann P.; Zivkovic A. M.; Zocco D.; Zuba-Surma E. K. Minimal Information for Studies of Extracellular Vesicles 2018 (MISEV2018): A Position Statement of the International Society for Extracellular Vesicles and Update of the MISEV2014 Guidelines. J. Extracell. Vesicles 2018. 10.1080/20013078.2018.1535750. PMC6322352 30637094

[ref49] Witwer K. W.; Goberdhan D. C. I.; O’Driscoll L.; Théry C.; Welsh J. A.; Blenkiron C.; Buzás E. I.; Di Vizio D.; Erdbrügger U.; Falcón-Pérez J. M.; Fu Q. L.; Hill A. F.; Lenassi M.; Lötvall J.; Nieuwland R.; Ochiya T.; Rome S.; Sahoo S.; Zheng L. Updating MISEV: Evolving the Minimal Requirements for Studies of Extracellular Vesicles. J. Extracell. Vesicles 2021, 10.1002/jev2.12182. PMC8710080 34953156

[ref50] Sung B. H.; von Lersner A.; Guerrero J.; Krystofiak E. S.; Inman D.; Pelletier R.; Zijlstra A.; Ponik S. M.; Weaver A. M. A Live Cell Reporter of Exosome Secretion and Uptake Reveals Pathfinding Behavior of Migrating Cells. Nat. Commun. 2020, 10.1038/s41467-020-15747-2. PMC7190671 32350252

[ref51] McInnes L.; Healy J.; Saul N.; Großberger L. UMAP: Uniform Manifold Approximation and Projection. J. Open Source Softw 2018, 3 (29), 861 10.21105/joss.00861.

[ref52] McInnes L.; Healy J.; Melville J. UMAP: Uniform Manifold Approximation and Projection for Dimension Reduction. arXiv, February 9, 2018. 10.48550/arXiv.1802.03426 (accessed on March 18, 2024).

[ref53] McInnes L.; Healy J. Accelerated Hierarchical Density Based Clustering. IEEE Int. Conf. Data Mining Workshops (ICDMW) 2017, 2017, 33–42. 10.1109/ICDMW.2017.12.

[ref54] Campello R. J. G. B.; Moulavi D.; Sander J. Density-Based Clustering Based on Hierarchical Density Estimates. In Advances in Knowledge Discovery and Data Mining; Springer: Berlin Heidelberg, 2013; pp 160–172.

[ref55] Clarke R. J.; Kane D. J. Optical Detection of Membrane Dipole Potential: Avoidance of Fluidity and Dye-Induced Effects. Biochimica et Biophysica Acta (BBA) - Biomembranes 1997, 1323 (2), 223–239. 10.1016/S0005-2736(96)00188-5. 9042345

[ref56] Andronico L. A.; Jiang Y.; Jung S. R.; Fujimoto B. S.; Vojtech L.; Chiu D. T. Sizing Extracellular Vesicles Using Membrane Dyes and a Single Molecule-Sensitive Flow Analyzer. Anal. Chem. 2021, 93 (14), 5897 10.1021/acs.analchem.1c00253. 33784071 PMC10243643

[ref57] Nolan J. P.; Duggan E. Analysis of Individual Extracellular Vesicles by Flow Cytometry. Methods Mol. Biol. 2018, 1678, 79–92. 10.1007/978-1-4939-7346-0_5. 29071676

[ref58] Van Der Pol E.; Van Gemert M. J. C.; Sturk A.; Nieuwland R.; Van Leeuwen T. G. Single vs. Swarm Detection of Microparticles and Exosomes by Flow Cytometry. J. Thromb Haemost 2012, 10 (5), 919–930. 10.1111/j.1538-7836.2012.04683.x. 22394434

[ref59] Löwe M.; Kalacheva M.; Boersma A. J.; Kedrov A. The More the Merrier: Effects of Macromolecular Crowding on the Structure and Dynamics of Biological Membranes. FEBS J. 2020, 287 (23), 5039–5067. 10.1111/febs.15429. 32463979

[ref60] Witwer K. W.; Buzás E. I.; Bemis L. T.; Bora A.; Lässer C.; Lötvall J.; Nolte-’t Hoen E. N.; Piper M. G.; Sivaraman S.; Skog J.; Théry C.; Wauben M. H.; Hochberg F. Standardization of Sample Collection, Isolation and Analysis Methods in Extracellular Vesicle Research. J. Extracell. Vesicles 2013, 10.3402/jev.v2i0.20360. PMC3760646 24009894

[ref61] Di Vizio D.; Kim J.; Hager M. H.; Morello M.; Yang W.; Lafargue C. J.; True L.; Rubin M. A.; Adam R. M.; Beroukhim R.; Demichelis F.; Freeman M. R. Oncosome Formation in Prostate Cancer: Association with a Region of Frequent Chromosomal Deletion in Metastatic Disease NIH Public Access. Cancer Res. 2009, 69 (13), 5601–5609. 10.1158/0008-5472.CAN-08-3860. 19549916 PMC2853876

[ref62] Kowal J.; Arras G.; Colombo M.; Jouve M.; Morath J. P.; Primdal-Bengtson B.; Dingli F.; Loew D.; Tkach M.; Théry C. Proteomic Comparison Defines Novel Markers to Characterize Heterogeneous Populations of Extracellular Vesicle Subtypes. Proc. Natl. Acad. Sci. U. S. A. 2016, 113 (8), E968–E977. 10.1073/pnas.1521230113. 26858453 PMC4776515

[ref63] Heimburg T. Phase Transitions in Biological Membranes. arXiv, May 29, 2018. 10.48550/arxiv.1805.11481 (accessed on March 18, 2024).

[ref64] Gaus K.; Gratton E.; Kable E. P. W.; Jones A. S.; Gelissen I.; Kritharides L.; Jessup W. Visualizing Lipid Structure and Raft Domains in Living Cells with Two-Photon Microscopy. Proc. Natl. Acad. Sci. U. S. A. 2003, 100 (26), 15554 10.1073/pnas.2534386100. 14673117 PMC307606

[ref65] Owen D. M.; Rentero C.; Magenau A.; Abu-Siniyeh A.; Gaus K. Quantitative Imaging of Membrane Lipid Order in Cells and Organisms. Nat. Protoc 2012, 7 (1), 24–35. 10.1038/nprot.2011.419. 22157973

[ref66] Oncul S.; Klymchenko A. S.; Kucherak O. A.; Demchenko A. P.; Martin S.; Dontenwill M.; Arntz Y.; Didier P.; Duportail G.; Mély Y. Liquid Ordered Phase in Cell Membranes Evidenced by a Hydration-Sensitive Probe: Effects of Cholesterol Depletion and Apoptosis. Biochimica et Biophysica Acta (BBA) - Biomembranes 2010, 1798 (7), 1436–1443. 10.1016/j.bbamem.2010.01.013. 20100458

[ref67] Kilin V.; Glushonkov O.; Herdly L.; Klymchenko A.; Richert L.; Mely Y. Fluorescence Lifetime Imaging of Membrane Lipid Order with a Ratiometric Fluorescent Probe. Biophys. J. 2015, 108 (10), 2521–2531. 10.1016/j.bpj.2015.04.003. 25992730 PMC4457243

[ref68] Robinson D.; Besley N. A.; O’Shea P.; Hirst J. D. Di-8-ANEPPS Emission Spectra in Phospholipid/Cholesterol Membranes: A Theoretical Study. J. Phys. Chem. B 2011, 115 (14), 4160–4167. 10.1021/jp1111372. 21425824

[ref69] Bouquiaux C.; Castet F.; Champagne B. Unravelling the Effects of Cholesterol on the Second-Order Nonlinear Optical Responses of Di-8-ANEPPS Dye Embedded in Phosphatidylcholine Lipid Bilayers. J. Phys. Chem. B 2021, 125 (36), 10195–10212. 10.1021/acs.jpcb.1c05630. 34491062

[ref70] Bondelli G.; Paternò G. M.; Lanzani G. INVITED) Fluorescent Probes for Optical Investigation of the Plasma Membrane. Optical Materials: X 2021, 12, 100085 10.1016/j.omx.2021.100085.

[ref71] Parasassi T.; De Stasio G.; d’Ubaldo A.; Gratton E. Phase Fluctuation in Phospholipid Membranes Revealed by Laurdan Fluorescence. Biophys. J. 1990, 57 (6), 1179 10.1016/S0006-3495(90)82637-0. 2393703 PMC1280828

[ref72] Bagatolli L. A.; Gratton E. Two-Photon Fluorescence Microscopy Observation of Shape Changes at the Phase Transition in Phospholipid Giant Unilamellar Vesicles. Biophys. J. 1999, 77 (4), 2090–2101. 10.1016/S0006-3495(99)77050-5. 10512829 PMC1300490

[ref73] Bagatolli L. A.; Gratton E. Two Photon Fluorescence Microscopy of Coexisting Lipid Domains in Giant Unilamellar Vesicles of Binary Phospholipid Mixtures. Biophys. J. 2000, 78 (1), 290 10.1016/S0006-3495(00)76592-1. 10620293 PMC1300637

[ref74] Demchenko A. P. Beyond Annexin V: Fluorescence Response of Cellular Membranes to Apoptosis. Cytotechnology 2013, 65 (2), 157 10.1007/s10616-012-9481-y. 22797774 PMC3560882

[ref75] Darwich Z.; Klymchenko A. S.; Kucherak O. A.; Richert L.; Mély Y. Detection of Apoptosis through the Lipid Order of the Outer Plasma Membrane Leaflet. Biochimica et Biophysica Acta (BBA) - Biomembranes 2012, 1818 (12), 3048–3054. 10.1016/j.bbamem.2012.07.017. 22846507

[ref76] Demchenko A. The Change of Cellular Membranes on Apoptosis: Fluorescence Detection. Exp. Oncol. 2012, 34 (3), 263–268. 23070011

[ref77] Shynkar V. V.; Klymchenko A. S.; Kunzelmann C.; Duportail G.; Muller C. D.; Demchenko A. P.; Freyssinet J. M.; Mely Y. Fluorescent Biomembrane Probe for Ratiometric Detection of Apoptosis. J. Am. Chem. Soc. 2007, 129 (7), 2187–2193. 10.1021/ja068008h. 17256940

[ref78] Sengupta S.; Karsalia R.; Morrissey A.; Bamezai A. K. Cholesterol-Dependent Plasma Membrane Order (Lo) Is Critical for Antigen-Specific Clonal Expansion of CD4+ T Cells. Scientific Reports 2021 11:1 2021, 11 (1), 1–9. 10.1038/s41598-021-93403-5. PMC8263698 34234214

[ref79] Nedbal J.; Visitkul V.; Ortiz-Zapater E.; Weitsman G.; Chana P.; Matthews D. R.; Ng T.; Ameer-Beg S. M. Time-Domain Microfluidic Fluorescence Lifetime Flow Cytometry for High-Throughput Förster Resonance Energy Transfer Screening. Cytometry Part A 2015, 87 (2), 104–118. 10.1002/cyto.a.22616. PMC4440390 25523156

[ref80] Osteikoetxea X.; Balogh A.; Szabó-Taylor K.; Németh A.; Szabó T. G.; Pálóczi K.; Sódar B.; Kittel Á.; György B.; Pállinger É.; Matkó J.; Buzás E. I. Improved Characterization of EV Preparations Based on Protein to Lipid Ratio and Lipid Properties. PLoS One 2015, 10 (3), e0121184 10.1371/journal.pone.0121184. 25798862 PMC4370721

[ref81] Oncul S.; Klymchenko A. S.; Kucherak O. A.; Demchenko A. P.; Martin S.; Dontenwill M.; Arntz Y.; Didier P.; Duportail G.; Mély Y. Liquid Ordered Phase in Cell Membranes Evidenced by a Hydration-Sensitive Probe: Effects of Cholesterol Depletion and Apoptosis. Biochimica et Biophysica Acta (BBA) - Biomembranes 2010, 1798 (7), 1436–1443. 10.1016/j.bbamem.2010.01.013. 20100458

[ref82] Owen D. M.; Williamson D. J.; Magenau A.; Gaus K. Sub-Resolution Lipid Domains Exist in the Plasma Membrane and Regulate Protein Diffusion and Distribution. Nature Communications 2012 3:1 2012, 3 (1), 1–8. 10.1038/ncomms2273. 23212385

[ref83] Pearson K. LIII. On Lines and Planes of Closest Fit to Systems of Points in Space. London, Edinburgh, Dublin Phil. Mag. J. Sci. 1901, 2 (11), 559–572. 10.1080/14786440109462720.

[ref84] Van Der Maaten L.; Hinton G. Visualizing Data Using T-SNE. J. Mach. Learn. Res. 2008, 9, 2579–2605.

[ref85] Huang H.; Wang Y.; Rudin C.; Browne E. P. Towards a Comprehensive Evaluation of Dimension Reduction Methods for Transcriptomic Data Visualization. Communications Biology 2022 5:1 2022, 5 (1), 1–11. 10.1038/s42003-022-03628-x. PMC9296444 35853932

[ref86] Berthold M. R.; Cebron N.; Dill F.; Gabriel T. R.; Kötter T.; Meinl T.; Ohl P.; Sieb C.; Thiel K.; Wiswedel B. KNIME: The Konstanz Information Miner. In Studies in Classification, Data Analysis, and Knowledge Organization (GfKL 2007); Springer, 2007.

[ref87] Fillbrunn A.; Dietz C.; Pfeuffer J.; Rahn R.; Landrum G. A.; Berthold M. R. KNIME for Reproducible Cross-Domain Analysis of Life Science Data. J. Biotechnol. 2017, 261, 149–156. 10.1016/j.jbiotec.2017.07.028. 28757290

[ref88] Gardiner C.; Di Vizio D.; Sahoo S.; Thery C.; Witwer K. W.; Wauben M.; Hill A. F. Techniques Used for the Isolation and Characterization of Extracellular Vesicles: Results of a Worldwide Survey. J. Extracell. Vesicles 2016, 10.3402/jev.v5.32945. PMC5090131 27802845

[ref89] Barone S. M.; Paul A. G. A.; Muehling L. M.; Lannigan J. A.; Kwok W. W.; Turner R. B.; Woodfolk J. A.; Irish J. M. Unsupervised Machine Learning Reveals Key Immune Cell Subsets in COVID-19, Rhinovirus Infection, and Cancer Therapy. eLife 2021, 10.7554/eLife.64653. PMC8370768 34350827

[ref90] Mathieu M.; Névo N.; Jouve M.; Valenzuela J. I.; Maurin M.; Verweij F. J.; Palmulli R.; Lankar D.; Dingli F.; Loew D.; Rubinstein E.; Boncompain G.; Perez F.; Théry C. Specificities of Exosome versus Small Ectosome Secretion Revealed by Live Intracellular Tracking of CD63 and CD9. Nat. Commun. 2021, 10.1038/s41467-021-24384-2. PMC8289845 34282141

[ref91] Hurwitz S. N.; Conlon M. M.; Rider M. A.; Brownstein N. C.; Meckes D. G. Nanoparticle Analysis Sheds Budding Insights into Genetic Drivers of Extracellular Vesicle Biogenesis. J. Extracell. Vesicles 2016, 10.3402/jev.v5.31295. PMC4947197 27421995

[ref92] Ostrowski M.; Carmo N. B.; Krumeich S.; Fanget I.; Raposo G.; Savina A.; Moita C. F.; Schauer K.; Hume A. N.; Freitas R. P.; Goud B.; Benaroch P.; Hacohen N.; Fukuda M.; Desnos C.; Seabra M. C.; Darchen F.; Amigorena S.; Moita L. F.; Thery C. Rab27a and Rab27b Control Different Steps of the Exosome Secretion Pathway. Nat. Cell Biol. 2010, 12 (1), 19–30. 10.1038/ncb2000. 19966785

[ref93] Verweij F. J.; Bebelman M. P.; Jimenez C. R.; Garcia-Vallejo J. J.; Janssen H.; Neefjes J.; Knol J. C.; de Goeij-de Haas R.; Piersma S. R.; Baglio S. R.; Verhage M.; Middeldorp J. M.; Zomer A.; van Rheenen J.; Coppolino M. G.; Hurbain I.; Raposo G.; Smit M. J.; Toonen R. F. G.; van Niel G.; Pegtel D. M. Quantifying Exosome Secretion from Single Cells Reveals a Modulatory Role for GPCR Signaling. J. Cell Biol. 2018, 217 (3), 1129–1142. 10.1083/jcb.201703206. 29339438 PMC5839777

[ref94] Hemler M. E. Tetraspanin Functions and Associated Microdomains. Nat. Rev. Mol. Cell Biol. 2005, 6 (10), 801–811. 10.1038/nrm1736. 16314869

[ref95] Levy S.; Shoham T. The Tetraspanin Web Modulates Immune-Signalling Complexes. Nat. Rev. Immunol 2005, 5 (2), 136–148. 10.1038/nri1548. 15688041

[ref96] Yáñez-Mó M.; Barreiro O.; Gordon-Alonso M.; Sala-Valdés M.; Sánchez-Madrid F. Tetraspanin-Enriched Microdomains: A Functional Unit in Cell Plasma Membranes. Trends in Cell Biology. 2009, 19, 434 10.1016/j.tcb.2009.06.004. 19709882

[ref97] Zijlstra A. Tetraspanins in Cancer. In Cell-Extracellular Matrix Interactions in Cancer; Springer, 2010; pp 217–243.

[ref98] Pols M. S.; Klumperman J. Trafficking and Function of the Tetraspanin CD63. Exp. Cell Res. 2009, 315 (9), 1584–1592. 10.1016/j.yexcr.2008.09.020. 18930046

[ref99] Zuidscherwoude M.; Göttfert F.; Dunlock V. M. E.; Figdor C. G.; Van Den Bogaart G.; Van Spriel A. B. The Tetraspanin Web Revisited by Super-Resolution Microscopy. Sci. Rep. 2015, 10.1038/srep12201. PMC4505338 26183063

[ref100] Fordjour F. K.; Guo C.; Ai Y.; Daaboul G. G.; Gould S. J. A Shared, Stochastic Pathway Mediates Exosome Protein Budding along Plasma and Endosome Membranes. J. Biol. Chem. 2022, 298 (10), 102394 10.1016/j.jbc.2022.102394. 35988652 PMC9512851

[ref101] Mariscal J.; Vagner T.; Kim M.; Zhou B.; Chin A.; Zandian M.; Freeman M. R.; You S.; Zijlstra A.; Yang W.; Di Vizio D. Comprehensive Palmitoyl-Proteomic Analysis Identifies Distinct Protein Signatures for Large and Small Cancer-Derived Extracellular Vesicles. J. Extracell. Vesicles 2020, 10.1080/20013078.2020.1764192. PMC7448892 32944167

[ref102] Vagner T.; Spinelli C.; Minciacchi V. R.; Balaj L.; Zandian M.; Conley A.; Zijlstra A.; Freeman M. R.; Demichelis F.; De S.; Posadas E. M.; Tanaka H.; Di Vizio D. Large Extracellular Vesicles Carry Most of the Tumour DNA Circulating in Prostate Cancer Patient Plasma. J. Extracell. Vesicles 2018, 10.1080/20013078.2018.1505403. PMC6084494 30108686

[ref103] Minciacchi V. R.; Spinelli C.; Reis-Sobreiro M.; Cavallini L.; You S.; Zandian M.; Li X.; Mishra R.; Chiarugi P.; Adam R. M.; Posadas E. M.; Viglietto G.; Freeman M. R.; Cocucci E.; Bhowmick N. A.; Di Vizio D. MYC Mediates Large Oncosome-Induced Fibroblast Reprogramming in Prostate Cancer. Cancer Res. 2017, 77 (9), 2306–2317. 10.1158/0008-5472.CAN-16-2942. 28202510

[ref104] Hope M. J.; Bally M. B.; Webb G.; Cullis P. R. Production of Large Unilamellar Vesicles by a Rapid Extrusion Procedure: Characterization of Size Distribution, Trapped Volume and Ability to Maintain a Membrane Potential. Biochim. Biophys. Acta 1985, 812 (1), 55–65. 10.1016/0005-2736(85)90521-8. 23008845

[ref105] Kotecha N.; Krutzik P. O.; Irish J. M. Web-Based Analysis and Publication of Flow Cytometry Experiments. Curr. Protoc. Cytom. 2010, 10.1002/0471142956.CY1017S53. PMC4208272 20578106

[ref106] Diggins K. E.; Greenplate A. R.; Leelatian N.; Wogsland C. E.; Irish J. M. Characterizing Cell Subsets Using Marker Enrichment Modeling. Nat. Methods 2017, 14 (3), 275–278. 10.1038/nmeth.4149. 28135256 PMC5330853

