# Supplementary material for: Multiparametric Single-Vesicle Flow Cytometry Resolves Extracellular Vesicle Heterogeneity and Reveals Selective Regulation of Biogenesis and Cargo Distribution
Source: ACS Nano. 2024 Apr 5;18(15):10464–84. doi: 10.1021/acsnano.3c11561 (PMC11025123; doi:10.1021/acsnano.3c11561)
Supplement: Supplementary file 1 — nn3c11561_si_001.pdf [file nn3c11561_si_001.pdf]

Supporting Information (SI)

Multi-parametric single-vesicle flow cytometry resolves extracellular vesicle heterogeneity and reveals selective regulation of biogenesis and cargo distribution

Authors: Ariana K. von Lersner[1], Fabiane Fernandes[2,3], Patricia Midori Murobushi Ozawa[4,5], Marques Jackson[6], Matthieu Masureel[7], Hoangdung Ho[7], Sierra M. Lima[5], Tatyana Vagner[8], Bong Hwan Sung[4,5], Mohamed Wehbe[9], Kai Franze[6,10], Heather Pua[2,4], John T. Wilson[1,2,4,9], Jonathan M. Irish[1,2,5], Alissa M. Weaver[1,2,4,5], Dolores Di Vizio[8*] and Andries Zijlstra[1,2,4,6*]

Affiliations:

[1]Program in Cancer Biology, Vanderbilt University, Nashville, 37232, TN, USA

[2]Department of Pathology, Microbiology and Immunology, Vanderbilt University Medical Center, Nashville, 37232, TN, USA

[3]Institute of Applied Biosciences and Chemistry, Hogeschool Arnhem en Nijmegen University of Applied Sciences, Nijmegen, 6525 EM, Gelderland, Netherlands

[4]The Center for EV Research, Vanderbilt University, Nashville, 37232, TN, USA

[5]Department of Cell and Developmental Biology, Vanderbilt University School of Medicine, Nashville, 37232, TN, USA

[6]Department of Research Pathology, Genentech, San Francisco, 94080, CA, USA

[7]Department of Structural Biology, Genentech, San Francisco, 94080, CA, USA

[8]Department of Surgery, Cedars-Sinai Medical Center, Los Angeles, 90048, CA, USA

[9]Department of Chemical and Biomolecular Engineering, Vanderbilt University, Nashville, 37232, TN, USA

[10]KNIME GmbH, Konstanz, 78467, Germany

* Co-correspondent authors

AZ (zijlstra.andries@gmail.com) and DDV (dolores.divizio@cshs.org)

Table of Contents:

Type	Description	Page
**Index**	Abbreviations List	3-4
**Table S1**	List of metrics collected by CellStream™.	5
**Table S2**	Validation of quantitative particle detection using flow cytometry.	6
**Figure S1**	Optimized assay conditions for detecting EV by flow cytometry with Di-8-ANEPPS.	7-8
**Table S3**	Summary of staining method conditions	9
**Figure S2**	Validation of quantitative particle detection using flow cytometry.	10-11
**Figure S3**	Spectral emissions of Di-8-ANEPPS reflect particle size and membrane order	12-13
**Table S4**	DSPC sizing liposome standards composition and size	14
**Table S5**	DSPC cholesterol liposome standards composition and size	14
**Figure S4**	Comparison of dimensional reduction approaches demonstrate optimal performance with UMAP	15
**Figure S5**	Semi-automated analysis pipeline for dimensional reduction and clustering of EV populations.	16-17
**Table S6**	EV Fingerprinting pipeline clustering parameters	18
**Figure S6**	Automated cluster-based gating is concordant with manual gating of synthetic sizing standards.	19-20
**Figure S7**	Sample selection criteria fitting for EV Fingerprinting.	21
**Figure S8**	Sizing characterization and di8 staining of DG-UC preps.	22-23
**Figure S9**	Rab27a KD specifically affects 100K EVs.	24-25
**Figure S10**	Parental and pHluorin-CD63 100K EV characterization and gating strategy.	26
**Figure S11**	Gating strategy for multiplex analysis of TSPANs.	27-29

Abbreviations List

100K	100,000 g
10K	10,000 g
2K	2,800 g
CD63-	CD63 negative
CD63+	CD63 positive
CD81+	CD81 positive
chol	Cholesterol
CM	Conditioned medium
DG-UC	Density gradient ultracentrifugation
Di8	Di-8-ANEPPS
di8+	di-8-ANEPPS positive
DMG-PEG2000	1,2-Dimyristoyl-rac- glycero-3-methoxypolyethylene glycol-2000
DOPC	1,2-dioleoyl-sn-glycero-3-phosphocholine
DPPC	dipalmitoylphosphatidylcholine
DSPC	1,2-distearoyl-sn-glycero-3-phosphocholine
dual+	dual positive
EE	Early endosome
EV	Extracellular vesicles
GP	Generalized polarization
HDBSCAN	Hierarchial density-based spatial clustering of applications with noise
KD	Knockdown
KD1	Knockdown 1
KD2	Knockdown 2
KNN	*k*-nearest neighbor
L-EV	Larger extracellular vesicles
Ld	Liquid disordered
Lo	Liquid ordered
MC	Molecular crowding
MFI	Median fluorescence intensity
MISEV	Minimal information for studies of EVs
MVB	Multivesicular bodies
NTA	Nanoparticle tracking analysis
PaCMAP	Pairwise controlled manifold approximation
Par	Parental
PCA	Principal component analysis
pHl	pHluorin-CD63
RT	Room temperature
S-EV	Smaller extracellular vesicles

Scr	Scrambled control
SSC	Side scatter
T-REX	Tracking responders expanding
t-SNE	t-distributed stochastic neighbor embedding
TDI-CCD	Time delay integration charge-coupled device
TEM	Transmission electron microscopy
tFL	Total median fluorescence
TRPS	Tunable resistive pulse sensing
TSPANs	Tetraspanins
UC	Ultracentrifugation
UMAP	Uniform manifold approximation projection

Table S1: List of metrics collected by CellStream™

Index	Feature Name	Feature type	Calculation
1	**Camera Timer**	Camera Metric	Digital shutter speed
2	**Camera Line Number**	Camera Metric	Rate at which exposure and readout can occur
3	**Time**	Instrument Metric	Time sample was collected, saved in meta data
4	**FlowSpeed**	Instrument Metric	Fluidics measurement of flow rate, saved in meta data
5	**Raw Max Pixel [Excitation nm /Emission nm /bandpass nm]**	Optical Metric	Maximum intensity in any pixel without background subtraction
6	**Raw Min Pixel [Excitation nm /Emission nm /bandpass nm]**	Optical Metric	Minimum intensity in any pixel without background subtraction
7	**Intensity [Excitation nm /Emission nm /bandpass nm]**	Optical Metric	Sum of all raw pixel intensity with background subtraction
8	**UCI [Excitation nm /Emission nm /bandpass nm]**	Optical Metric	Uncompensated intensity, same measure as Intensity since we do not perform compensation
9	**Mean Pixel [Excitation nm /Emission nm /bandpass nm]**	Optical Metric	Mean pixel Intensity after background subtraction
10	**Bkgd Mean [Excitation nm /Emission nm /bandpass nm]**	Optical Metric	Mean background pixel intensity
11	**Bkgd StdDev [Excitation nm /Emission nm /bandpass nm]**	Optical Metric	Standard deviation of background intensity
12	**Area [Excitation nm /Emission nm /bandpass nm]**	Optical Metric	Pixel area of captured signal
13	**Gradient RMS [Excitation nm /Emission nm /bandpass nm]**	Optical Metric	Mean slope across three pixels, measure of image contrast and focus quality
14	**Major Axis [Excitation nm /Emission nm /bandpass nm]**	Optical Metric	Longest axis of particle
15	**Minor Axis [Excitation nm /Emission nm /bandpass nm]**	Optical Metric	Shortest axis of particle
16	**Aspect Ratio [Excitation nm /Emission nm /bandpass nm]**	Optical Metric	Aspect Ratio of particle
17	**Saturation Count [Excitation nm /Emission nm /bandpass nm]**	Optical Metric	Number of pixels saturated
18	**Saturation Percent [Excitation nm /Emission nm /bandpass nm]**	Optical Metric	Percentage of pixels saturated

Table S2: List of available features and which were used in UMAP and clustering

Index	Feature Name	Fluorochrome	Ex (nm)	Calculation	Em (nm)	Used in Initial Analysis?	Used in Re-analysis?
1	488 - 528	Di-8-ANEPPS	488	Intensity	528	Yes	Yes
2	RawMaxPixel_488 - 528	Di-8-ANEPPS	488	Raw Max Pixel	528	Yes	Yes
3	Area_488 - 528	Di-8-ANEPPS	488	Area	528	Yes	Yes
4	AspectRatio_488 - 528	Di-8-ANEPPS	488	AspectRatio	528	Yes	Yes
5	488 - 583	Di-8-ANEPPS	488	Intensity	583	Yes	Yes
6	RawMaxPixel_488 - 583	Di-8-ANEPPS	488	Raw Max Pixel	583	Yes	Yes
7	Area_488 - 583	Di-8-ANEPPS	488	Area	583	Yes	Yes
8	AspectRatio_488 - 583	Di-8-ANEPPS	488	AspectRatio	583	Yes	Yes
9	488 - 611	Di-8-ANEPPS	488	Intensity	611	Yes	Yes
10	RawMaxPixel_488 - 611	Di-8-ANEPPS	488	Raw Max Pixel	611	Yes	Yes
11	Area_488 - 611	Di-8-ANEPPS	488	Area	611	Yes	Yes
12	AspectRatio_488 - 611	Di-8-ANEPPS	488	AspectRatio	611	Yes	Yes
13	488 - 702	Di-8-ANEPPS	488	Intensity	702	Yes	Yes
14	RawMaxPixel_488 - 702	Di-8-ANEPPS	488	Raw Max Pixel	702	Yes	Yes
15	Area_488 - 702	Di-8-ANEPPS	488	Area	702	Yes	Yes
16	AspectRatio_488 - 702	Di-8-ANEPPS	488	AspectRatio	702	Yes	Yes
17	488 - 773	Di-8-ANEPPS	488	Intensity	773	Yes	Yes
18	RawMaxPixel_488 - 773	Di-8-ANEPPS	488	Raw Max Pixel	773	Yes	Yes
19	Area_488 - 773	Di-8-ANEPPS	488	Area	773	Yes	Yes
20	AspectRatio_488 - 773	Di-8-ANEPPS	488	AspectRatio	773	Yes	Yes
21	642-702	anti-CD63-APC	642	Intensity	702	No	Yes
22	RawMaxPixel_642-702	anti-CD63-APC	642	Raw Max Pixel	702	No	Yes
23	Area_642-702	anti-CD63-APC	642	Area	702	No	Yes
24	AspectRatio_642-702	anti-CD63-APC	642	AspectRatio	702	No	Yes
25	488 - 528	CD63-pHluorin	488	Intensity	528	Yes	Yes
26	RawMaxPixel_488 - 528	CD63-pHluorin	488	Raw Max Pixel	528	Yes	Yes
27	Area_488 - 528	CD63-pHluorin	488	Area	528	Yes	Yes
28	AspectRatio_488 - 528	CD63-pHluorin	488	AspectRatio	528	Yes	Yes

Optimized assay conditions for detecting EV by flow cytometry with Di-8-ANEPPS

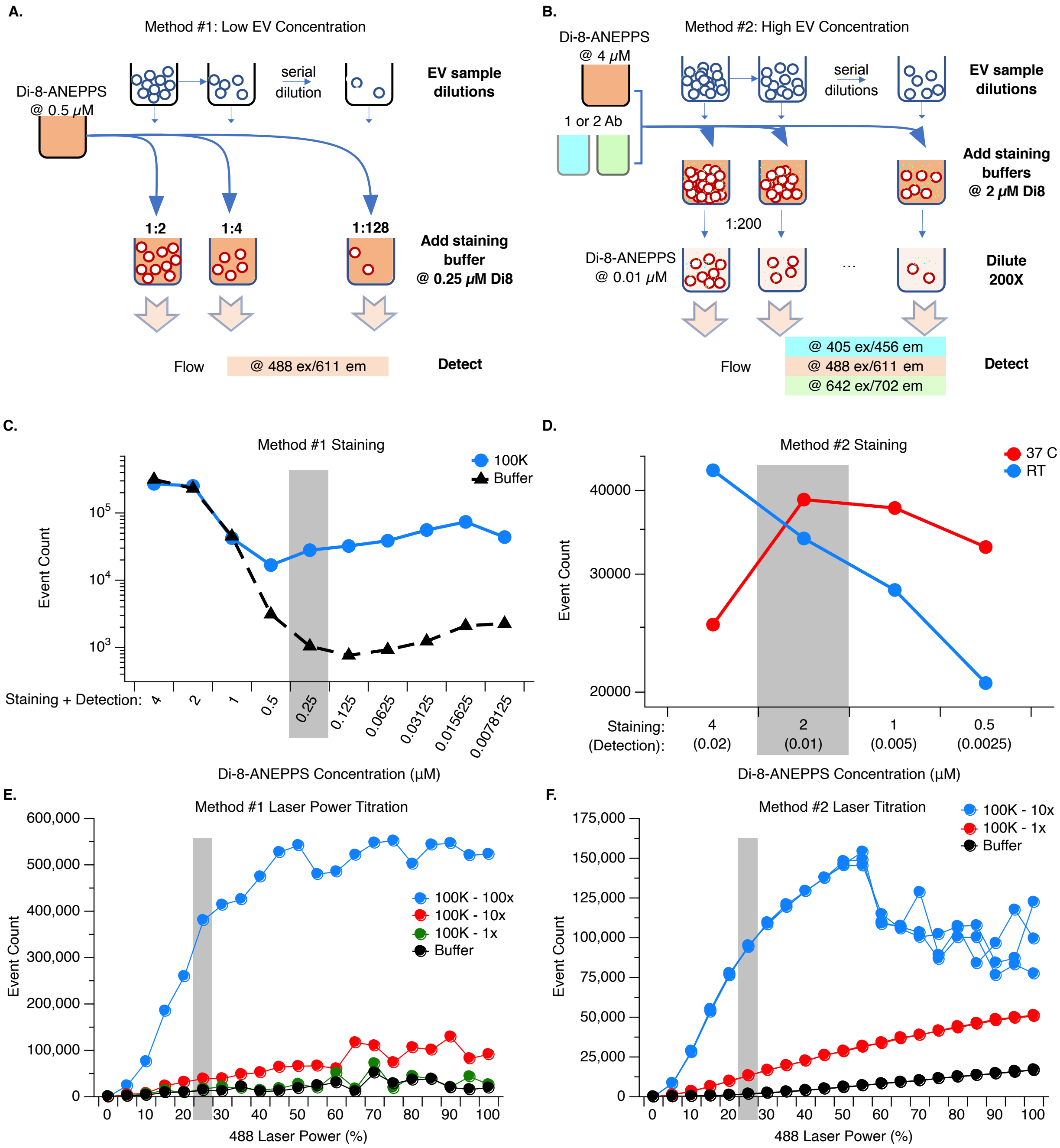

Figure S1. (A, B) Schematic of di8 staining of EVs using Method #1, employed for EV samples of low concentration without post-stain dilution **(A),** and Method #2, used for concentrated EV samples which includes a 200-fold post-stain dilution **(B)**. **(C)** Identification of optimal di8 concentration of staining + detection in Method #1 by comparing the detection of HT1080 EVs (100K) (blue circles) vs. buffer control (black triangles) across a 2-fold serial titration. Gray box indicates selected concentration. **(D)** Identification of optimal di8 staining concentrating and

temperature for Method #2 by evaluating optimal detection of HT1080 100K EVs upon 2-fold serial titration of di8. Gray box indicates selected concentration. Data was collected for 2 min. **(E,F)** Evaluation of optimal laser excitation power for Method #1 and #2 using di8 stained HT1080 100k preparation at 5% intervals. Optimal range is indicated in gray. For each experiment the events were collected for 2 min. Data shown are absolute counts from (n = 3) repeated reads. **(E)** Method #1 staining at 0.25 μM di8 and evaluation of three serial 10-fold dilutions. **(F)** Method #2 staining at 0.25 μM di8 and evaluation of two serial 10-fold dilutions.

Table S3: Summary of staining method conditions

Method:	Sample Concentration:	Di8 Staining Concentration (μM):	200x Post-Stain dilution?	Final Di8 Concentration:	Staining Conditions:	MC Buffer Stock:	488 Laser Power:
1	Low (Ex: CM, dilute EV preps)	0.25 μM	no	0.25 μM	1 hr RT	6.5%	25%
2	High (Ex: 10K and 100K EVs, plasma)	2 μM	yes	0.01 μM	1 hr (-ab) 3 hr (+ab) Both @ 37 C	6.5%	25%

Validation of quantitative particle detection using flow cytometry

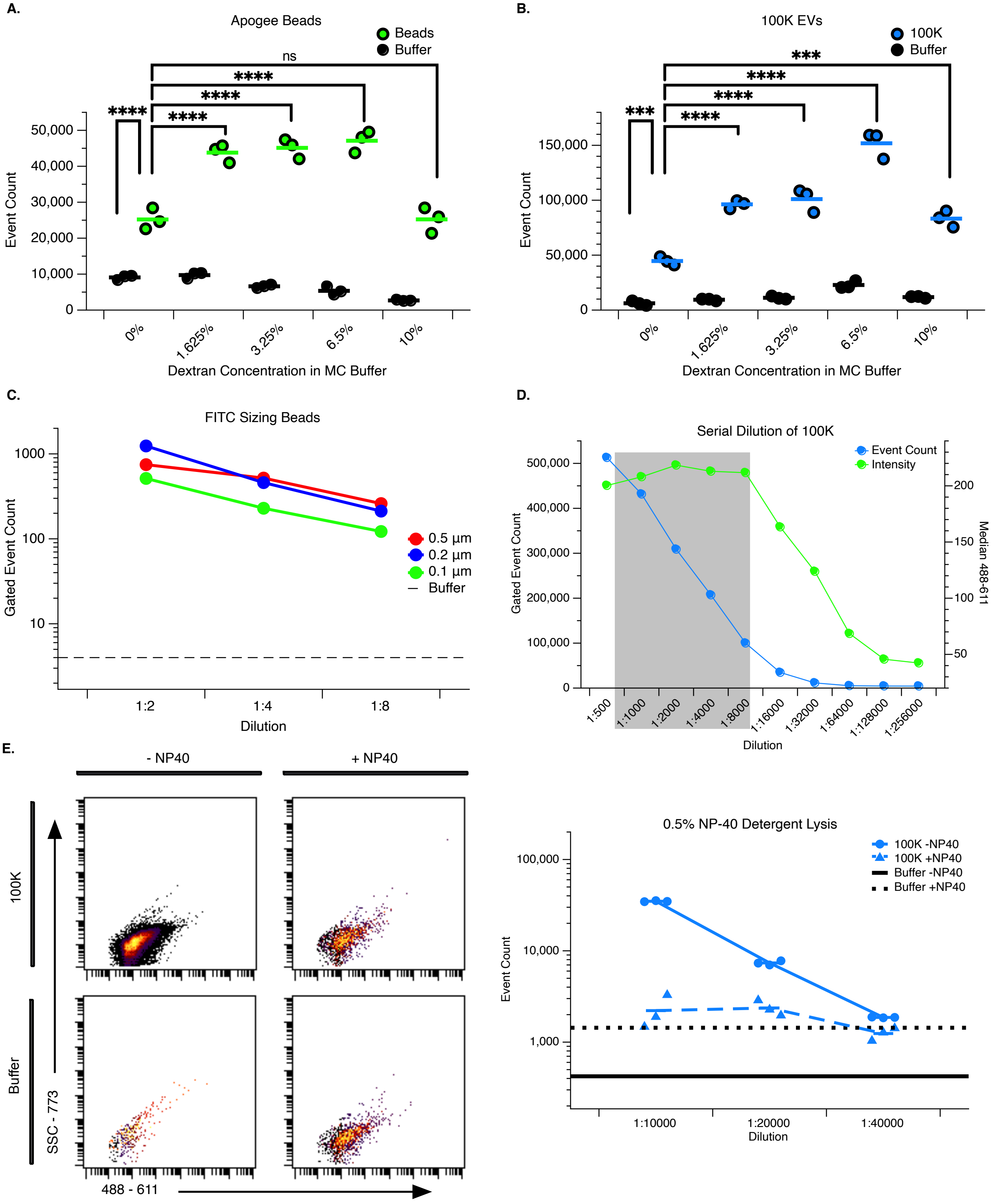

Figure S2. (A,B) Optimization of molecular crowding buffer (MC) for the detection of Apogee beads **(A)** and EVs **(B)** using increasing concentrations of dextran. Raw event counts for (n = 3) technical replicates are plotted for each sample type. Data were collected for 2 min and 488-laser

power was set to 25%. 2-way ANOVA with multiple comparisons, *** = P=0.0003, **** = P<0.0001 **(C)** Quantitative detection of FITC-labeled sizing beads (red, blue, and green solid) across serial dilutions of beads and buffer control (black dotted). Beads were diluted in MC buffer (3.25% dextran). Data collected at 25% power of 488 nm laser for 2 min. Absolute counts from (n = 1) representative experiment. **(D)** Establishing the quantitative detection range for single EV Flow cytometry with di8 stained HT1080 100K EV across 2-fold serial dilutions. Event count (blue) is plotted on the left y-axis and di8 median fluorescence intensity (green) is plotted on the right y-axis. Representative data shown for (n = 1) biological replicate. Samples were collected for 2 minutes. The quantitative range is indicated with a gray box. **(E)** Loss of EV detection upon lysis with 0.5% NP-40. Representative flow cytometry plots (left) of di8 stained HT1080 100K EVs (blue) and buffer (black) with (+) and without (-) NP-40. Absolute counts for (n = 3) technical replicates. Data were collected for 1 min.

Spectral emissions of Di-8-ANEPPS reflect particle size and membrane order

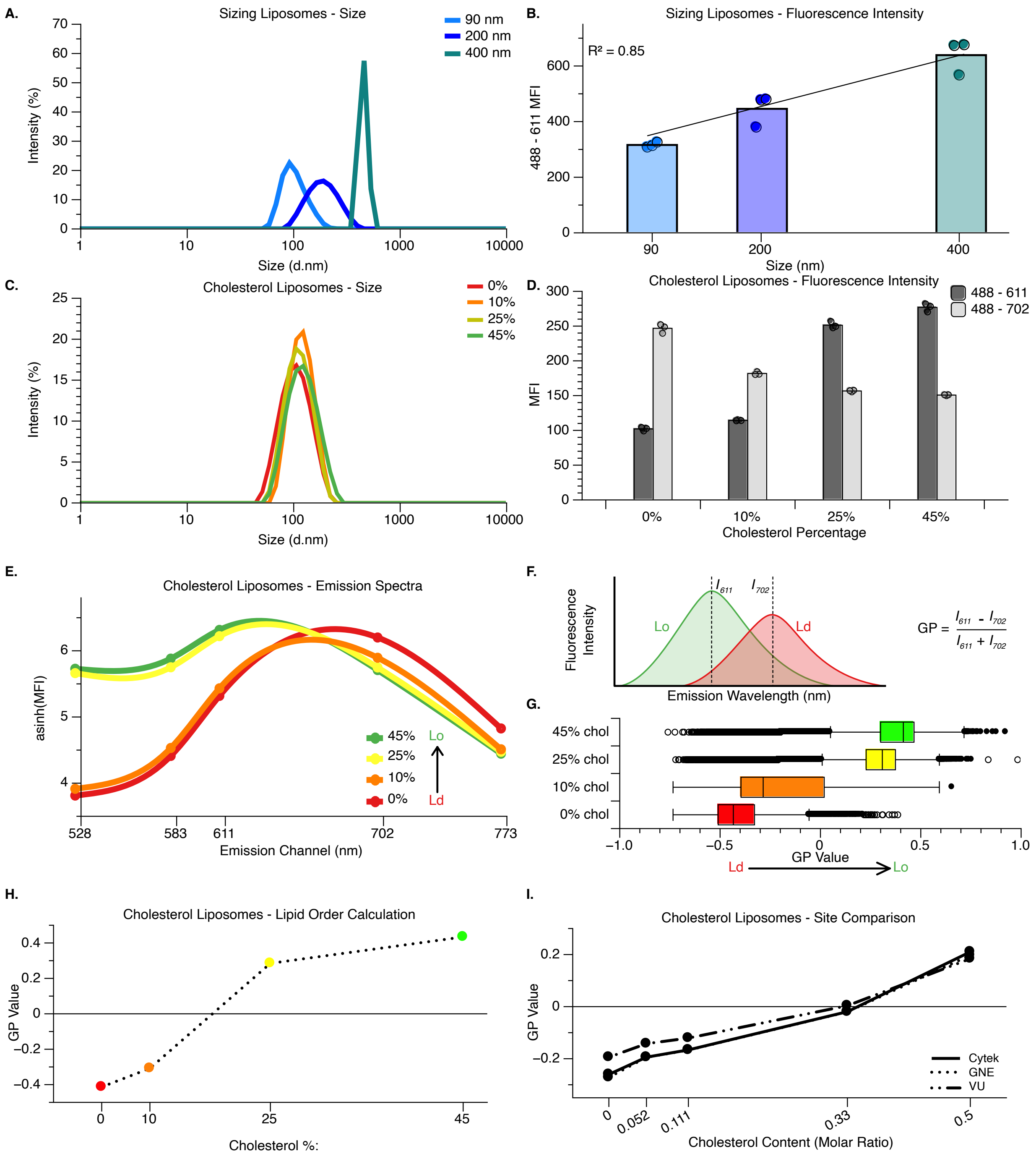

Figure S3. (A) Dynamic light scattering (DLS) intensity showing size distribution of 1,2-Distearoyl-sn-glycero-3-phosphocholine (DSPC) based sizing liposomes (90 nm, 200 nm, and 400 nm). **(B)** Fluorescence characterization of DSPC sizing liposomes from (A) using di8 (488-611) median fluorescence intensity (MFI) by flow cytometry. Data shown are from (n = 3) technical replicates collected for 5 min. Linear regression analysis: $R^2 = 0.85$. **(C-I)** Evaluation of membrane

order standards comprised of DSPC, cholesterol (chol), and 1,2-Dimyristoyl-rac- glycero-3-methoxypolyethylene glycol-2000 (DMG-PEG2000) liposomes generated with increasing amounts of cholesterol generating shifts from highly disordered membrane (Ld, 0% cholesterol) to highly ordered membrane (Lo, 45% cholesterol). **(C)** DLS intensity sizing determination of DSPC/chol/DMG-PEG2000 membrane order standards. **(D)** Fluorescence characterization of DSPC/chol/DMG-PEG2000 standards by flow cytometry for di8 peak emission at 611 nm and 702 nm after excitation at 488 nm. Data was collected for 5 min. **(E)** Emission spectra of di8 stained DSPC/chol/DMG-PEG2000 membrane order standards mapped across all 5 emission channels to visualize shifts in response to cholesterol-mediated increase in membrane order (red to green). Data shown are from (n = 1) technical replicate and collected for 2 min. **(F)** Graphical representation of the spectral shift associated with an increase in membrane order and the generalized polarization (GP) calculation as a simplified metric. For di8, fluorescence intensity (I) at emissions of 611 (I_{611}) and 702 (I_{702}). **(G)** Box plot of GP values of DSPC/chol/DMG-PEG2000 membrane order standards calculated from **G**. **(H)** Line plot of median GP values of DSPC/chol/DMG-PEG2000 membrane order standards. for cholesterol-containing liposomes stained with di8 collected in **G**. **(I)** Cross-institutional evaluation of GP values for DOPC-based membrane standards analyzed at three different sites: Cytek (solid line), Genentech (GNE, dotted line), and Vanderbilt University (VU, dotted solid line). Data were collected for 2 min. Average GP values from (n = 3) technical replicates are shown.

Table S4: DSPC sizing liposome standards composition and size

Sample	Composition	Lipid Ratios	Z-Maximum (nm)
400 (SUVs)	DSPC/Chol/DMG-PEG_{2000}	(52:45:3)	458.67
200 (SUVs)	DSPC/Chol/DMG-PEG_{2000}	(52:45:3)	190.14
90 (SUVs)	DSPC/Chol/DMG-PEG_{2000}	(52:45:3)	91.28

Table S5: DSPC cholesterol liposome standards composition and size

Sample	Composition	Lipid Ratios	Z-Maximum (nm)
0% Cholesterol	DSPC/DMG-PEG_{2000}	(95:5)	105.71
10% Cholesterol	DSPC/Chol/DMG-PEG_{2000}	(87:10:3)	122.42
25% Cholesterol	DSPC/Chol/DMG-PEG_{2000}	(72:25:3)	105.71
45% Cholesterol	DSPC/Chol/DMG-PEG_{2000}	(52:45:3)	122.42

Comparison of dimensional reduction approaches demonstrate optimal performance with UMAP

A.

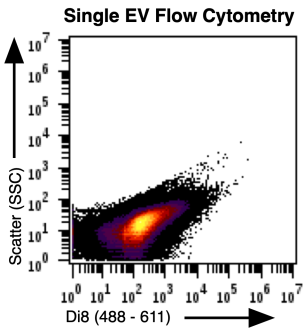

B.

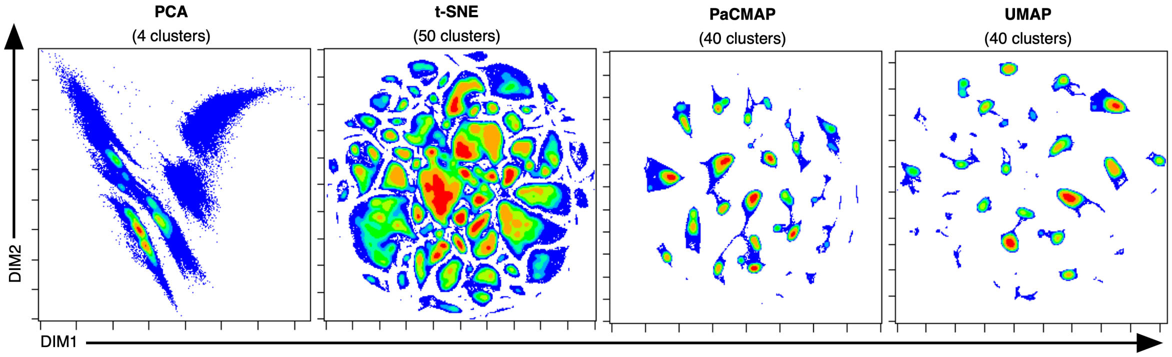

C.

Method	Number of Clusters	Unclassified Events (-1)	Di8 Range (488 - 611) Min/Max	tFL Range Min/Max	GP Range Min/Max	Unique UMAP Cluster Match (%)
UMAP	40	3098	11.8 / 3898	16 / 4634	-0.477 / +0.337	100
PaCMAP	40	18588	11.8 / 7879	16 / 9671	-0.476 / +0.341	92
T-SNE	50	24405	6.7 / 4154.4	10 / 4942	-0.533 / +0.336	80
PCA	4	0	136 / 1862	165 / 2177	-0.128 / -0195	nt

Figure S4. (A) Flow cytometry scatter plot of di8 stained HT1080 100K UC EV preparation gated for di8 positive events. **(B)** Resulting embeddings of dimensional reduction algorithms applied to A using PCA, t-SNE, PaCMAP, and UMAP **(C)** Table of metrics comparing dimensional reductions algorithms from B after applying the clustering algorithm HDBSCAN and extracting median features from each cluster. "nt" not tested

Semi-automated analysis pipeline for dimensional reduction and clustering of EV populations

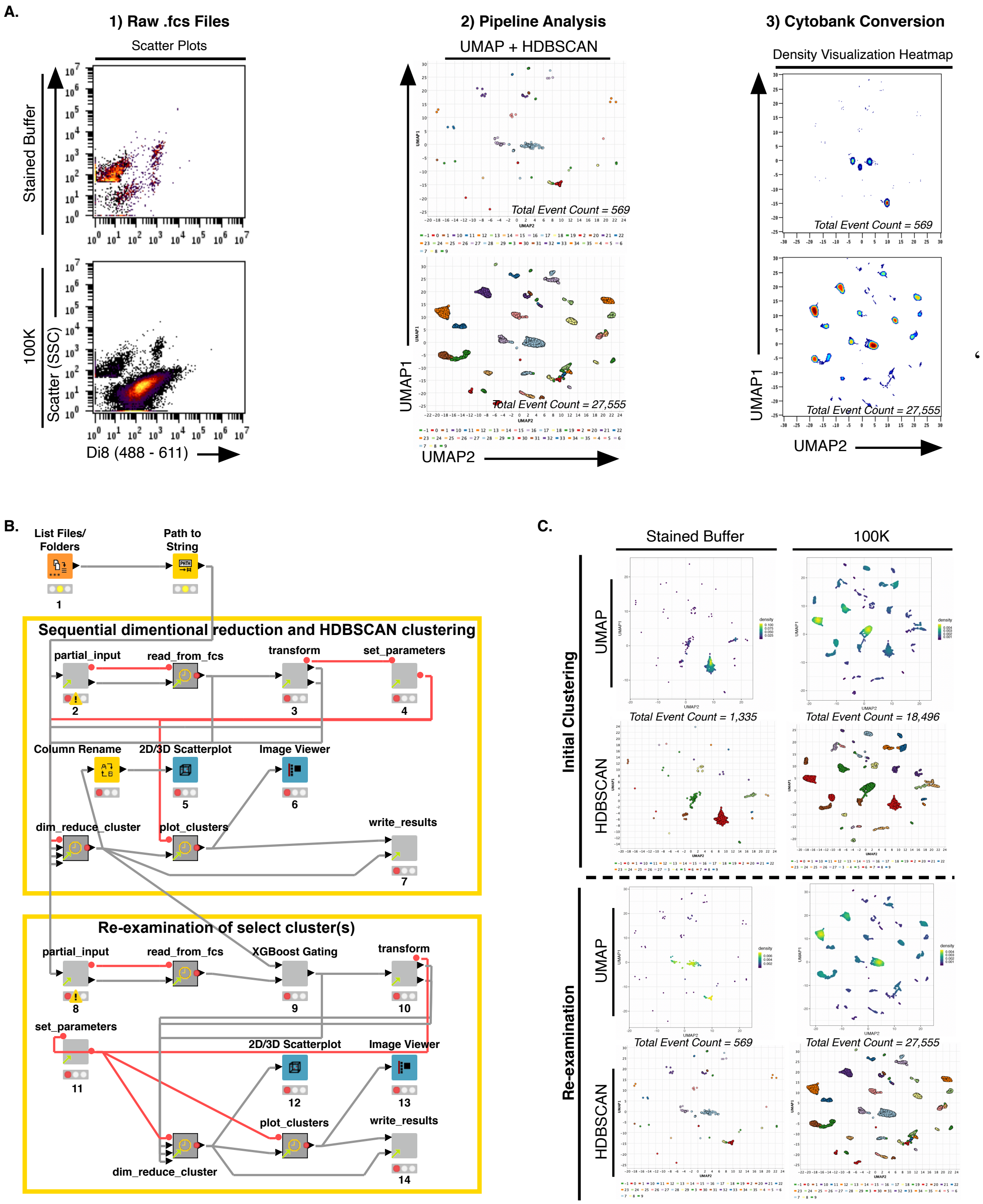

Figure S5. (A) Workflow of data analysis and processing after collection. Raw flow cytometry data (left, .fsc files**)** are uploaded and analyzed using the EV Fingerprinting pipeline in KNIME. Data are reduced in dimension (UMAP) and cluster identified (HDBSCAN) then output is exported as .csv files (middle). The .csv files are uploaded to Cytobank for final visualization (right). **(B)** The EV Fingerprinting pipeline in KNIME. Each node performs a data processing step

that is sequentially executed to generate results. The pipeline is split into two parts: Initial clustering (“Sequential dimensional reduction and HDBSCAN clustering”, top) and re-examination (“Re-examination of select cluster(s)”, bottom). **(C)** Resulting data from Method #2 stained of an HT1080 100K EV sample with non-specific buffer cluster removal. Raw .fcs files from a 100K EV sample and buffer control were analyzed with 50% relative sampling and the stained buffer control cluster was identified (top panels). Re-examination of all remaining clusters, with the exception of the non-specific buffer control cluster, was performed with 80% relative sampling (bottom panels).

Table S6: EV Fingerprinting pipeline clustering parameters

Clustering Parameter (“set_parameters” node):	HDBSCAN min_cluster size	UMAP n_neighbors	UMAP n_epochs	HDBSCAN min_samples	UMAP min_dist
Set Value:	1000	15	1,000	500	0.1

Automated cluster-based gating is concordant with manual gating of synthetic sizing standards

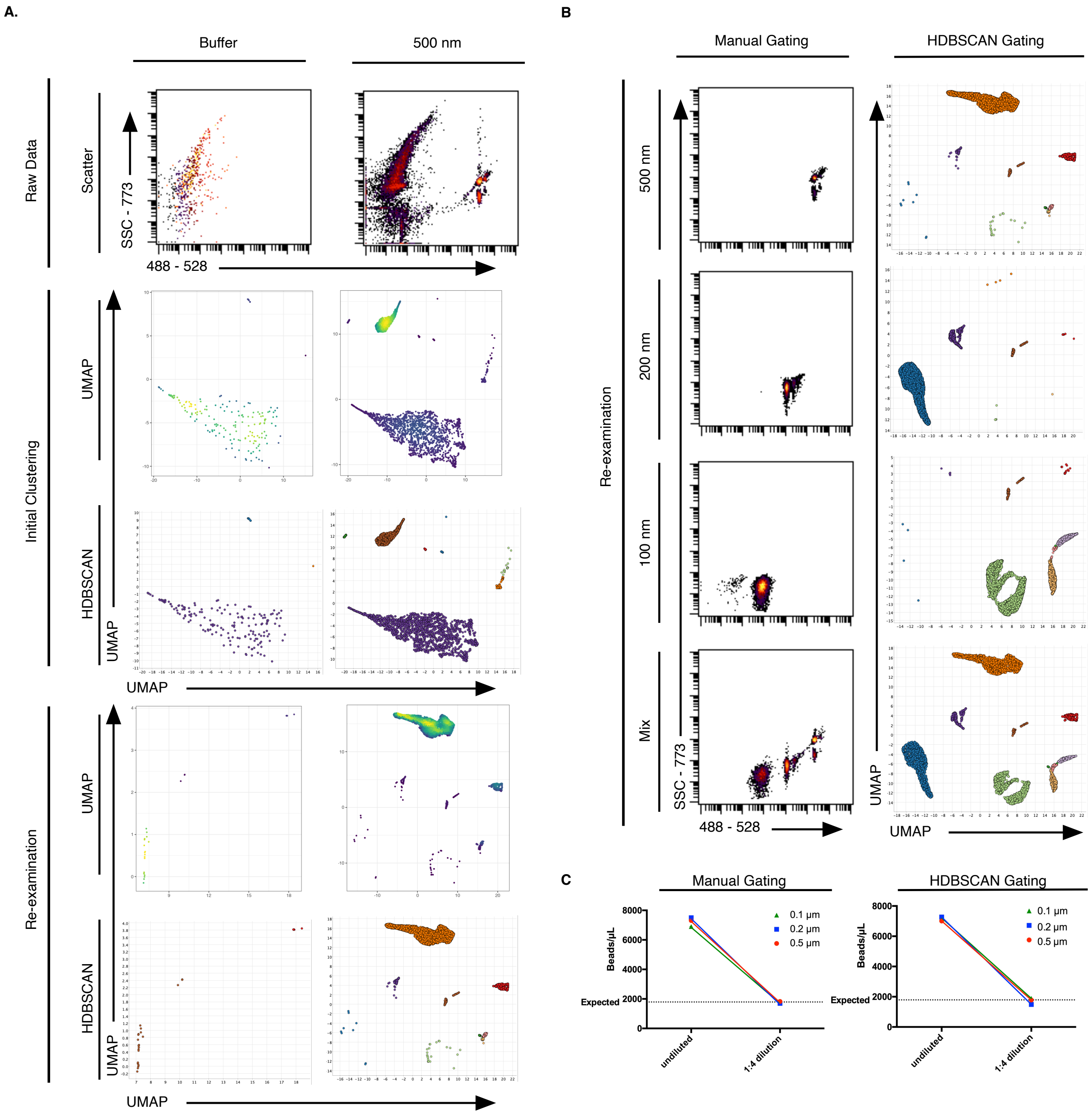

Figure S6. (A) Strategy for cluster based gating of a non-specific cluster from a blank buffer control. Flow cytometry of FITC labeled sizing beads (500 nm) were acquired with 100% 488-laser power for 2 minutes (top panels). Data were initially analyzed in the EV Fingerprinting pipeline with 80% relative sampling with a non-stained buffer control ("Initial Clustering, middle panels). The buffer-specific cluster was removed and re-examination was performed with 90% relative sampling. **(B)** Manual gating (left column) and cluster based HDBSCAN gating (right

column) of individual FITC sizing beads (500 nm, 200 nm, and 100 nm) and a mixture (MIX) of all beads. **(C)** Quantitative comparison of manual gating (left) and cluster based HDBSCAN gating (right) of FITC sizing beads across two dilutions. Dotted line indicates expected value from manufacturer at 1900 beads/µL. Data shown are from (n = 1) technical replicates.

Sample selection criteria fitting for EV Fingerprinting

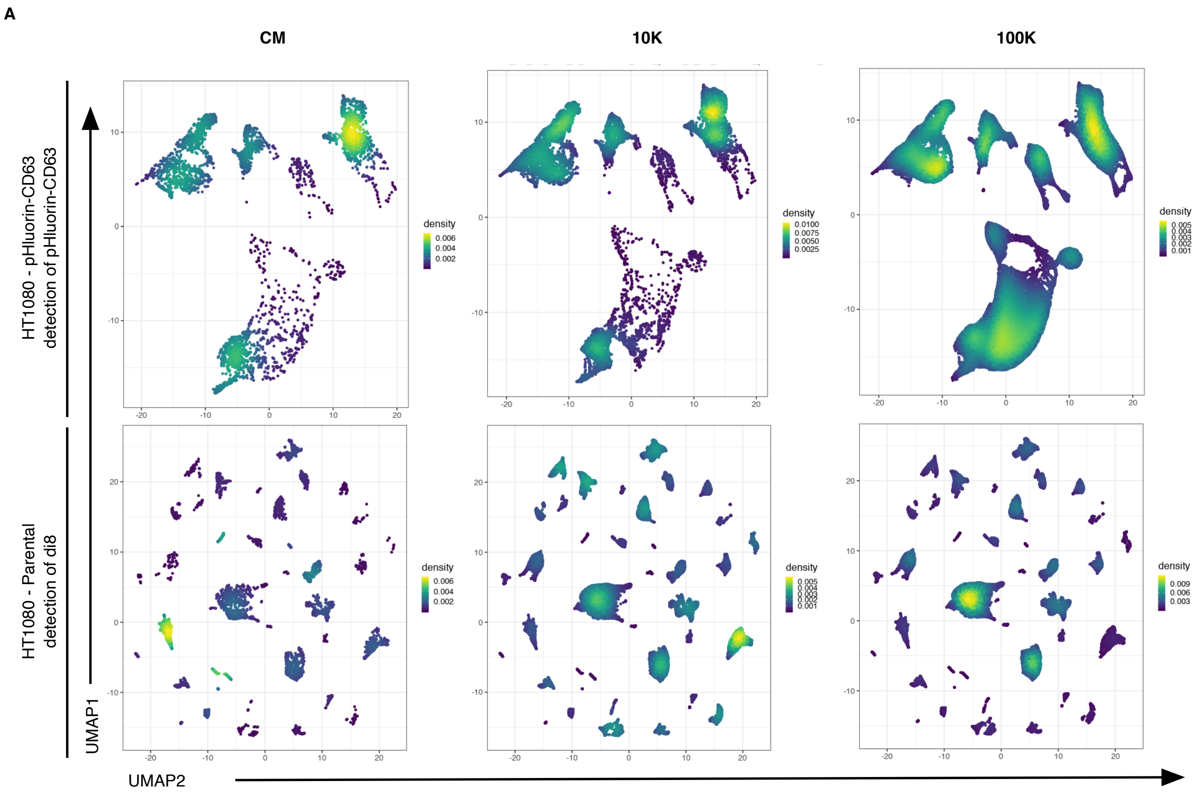

Figure S7. (A) Scatter plots of UMAP embeddings for UC EV preparations from HT1080-pHluorin, triggered for intrinsic pHluorin-CD63 signal, without di8 staining (Top panels) and HT1080-Parental cells stained with di8 (Bottom panels). Dimensional reduction was performed with emission features from 488 excitation laser (Supplementary Table 2).

Sizing characterization and di8 staining of DG-UC preps

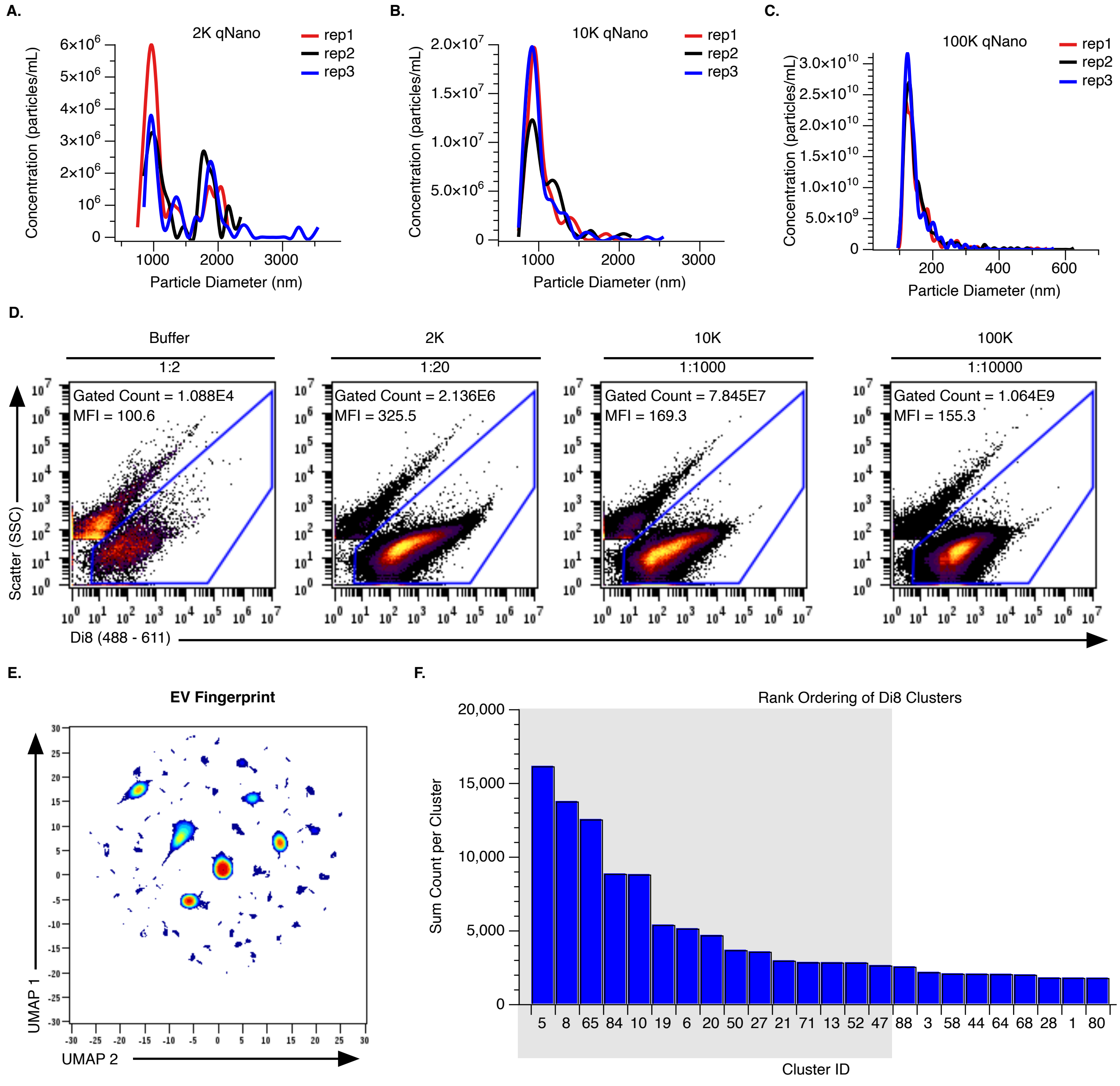

Figure S8. (A-C) Tunable resistive pulse sensing (TRPS) measurements showing size distribution and particle concentration of density gradient ultracentrifugation (DG-UC) isolated 2,800 g (2K, **A**), 10,000 g (10K, **B**) and 100,000 g (100K, **C)** from PC3 cells. Each sample measurement was taken as three technical replicates (reps 1-3) after fresh isolation. **(D)** Representative flow cytometry scatter plots of blank buffer and DG-UC isolated PC3 EVs (2K, 10K, and 100K) stained with di8 after freeze/thaw at -80°C. 2K and buffer control samples were stained using Method #1 (low particle concentration) while 10K and 100K samples were stained using Method #2 (high particle concentration). Data was collected for 10 min using EV Fingerprinting acquisition settings. Particle positive gating is shown (blue). Gated counts shown are dilution corrected. 488-611

median fluorescence intensities (MFI) by sample are depicted. **(E)** Representative EV Fingerprint of di8-positive events in EVs with 50% relative sampling for a total of 196,808 events. **(F)** Clusters from **E** ranked by abundance. The top 15 clusters were used for re-examination.

Rab27a KD specifically affects 100K EVs

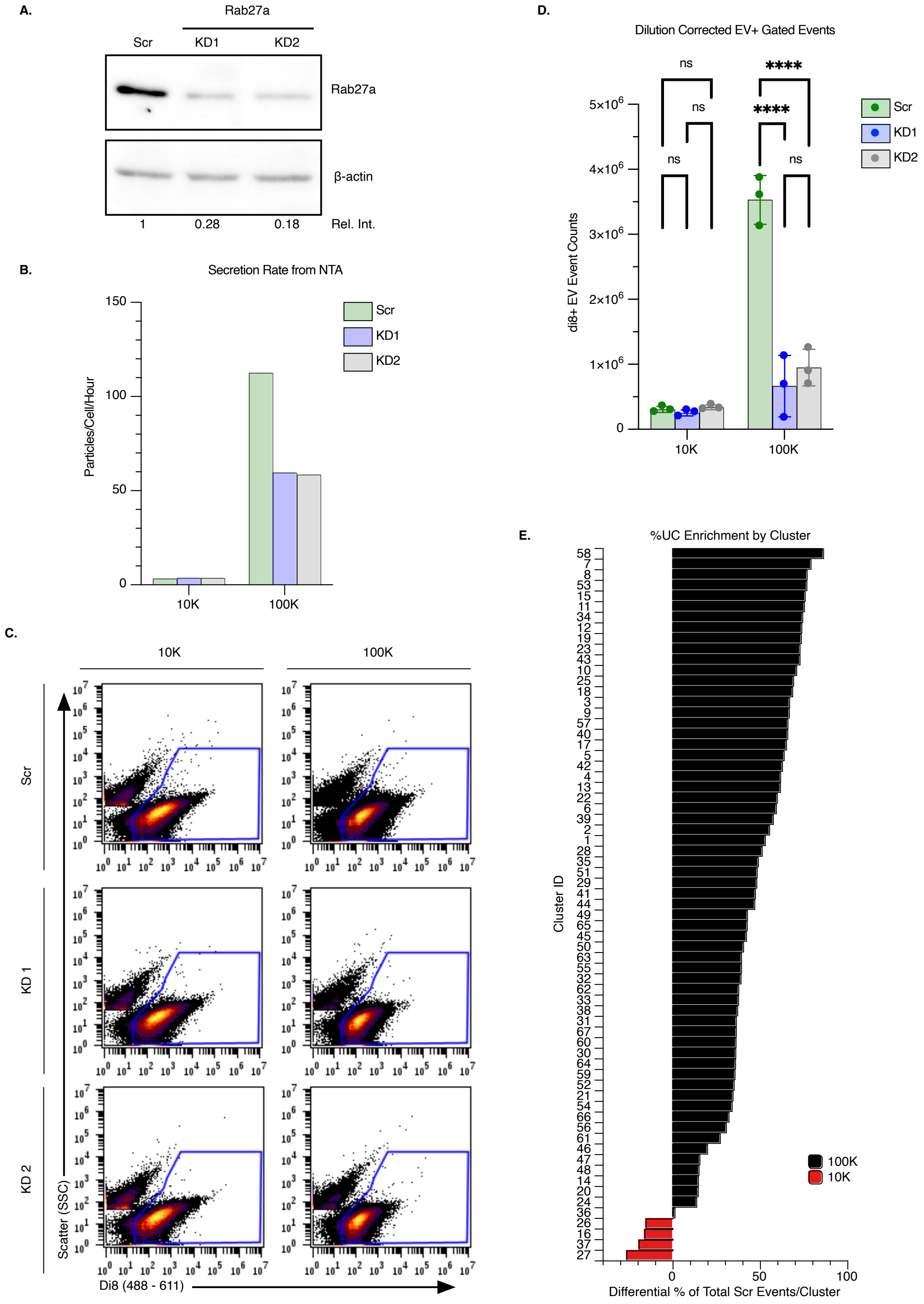

Figure S9. (A) Western blotting analysis confirming knock down (KD) of Rab27a protein in HT1080-Scr (Scr) cells compared to HT1080-KD1 (KD1) and HT1080-KD2 (KD2). **(B)** EV secretion rate as EVs secreted per cell per hour using nanoparticle tracking analysis (NTA) data from Scr and KD1/KD2 differential ultracentrifugation purified (d-UC) 10K and 100K EV after fresh isolation. Data shown is from (n = 1) biological replicate. **(C)** Representative scatter plots depicting flow cytometry gating strategy from Method #2 stained Scr and KD1/KD2 d-UC 10K and 100K EV after storage at 4°C for less than one week. The gate with di8 positive events selected as "EVs" is shown in blue. Dilution corrected event counts from (n = 3) technical replicates for 10 min using EV Fingerprinting acquisition settings. **(D)** Quantitative comparison of EV secretion from 10K and 100K EV from Scr and KD1/KD2 cells by flow cytometry. Technical replicates from three serial dilutions are plotted and the number of EVs was corrected for dilution. **** P<0.0001 by two-way ANOVA test. **(E)** Enrichment of events per cluster in the 10K or 100K prep quantified as the difference in percent of total from the Scr. Cluster_IDs are represented by decreasing enrichment in the 100K (black) and subsequent increasing enrichment in the 10K (red) from top to bottom.

Parental and pHluorin-CD63 100K EV characterization and gating strategy

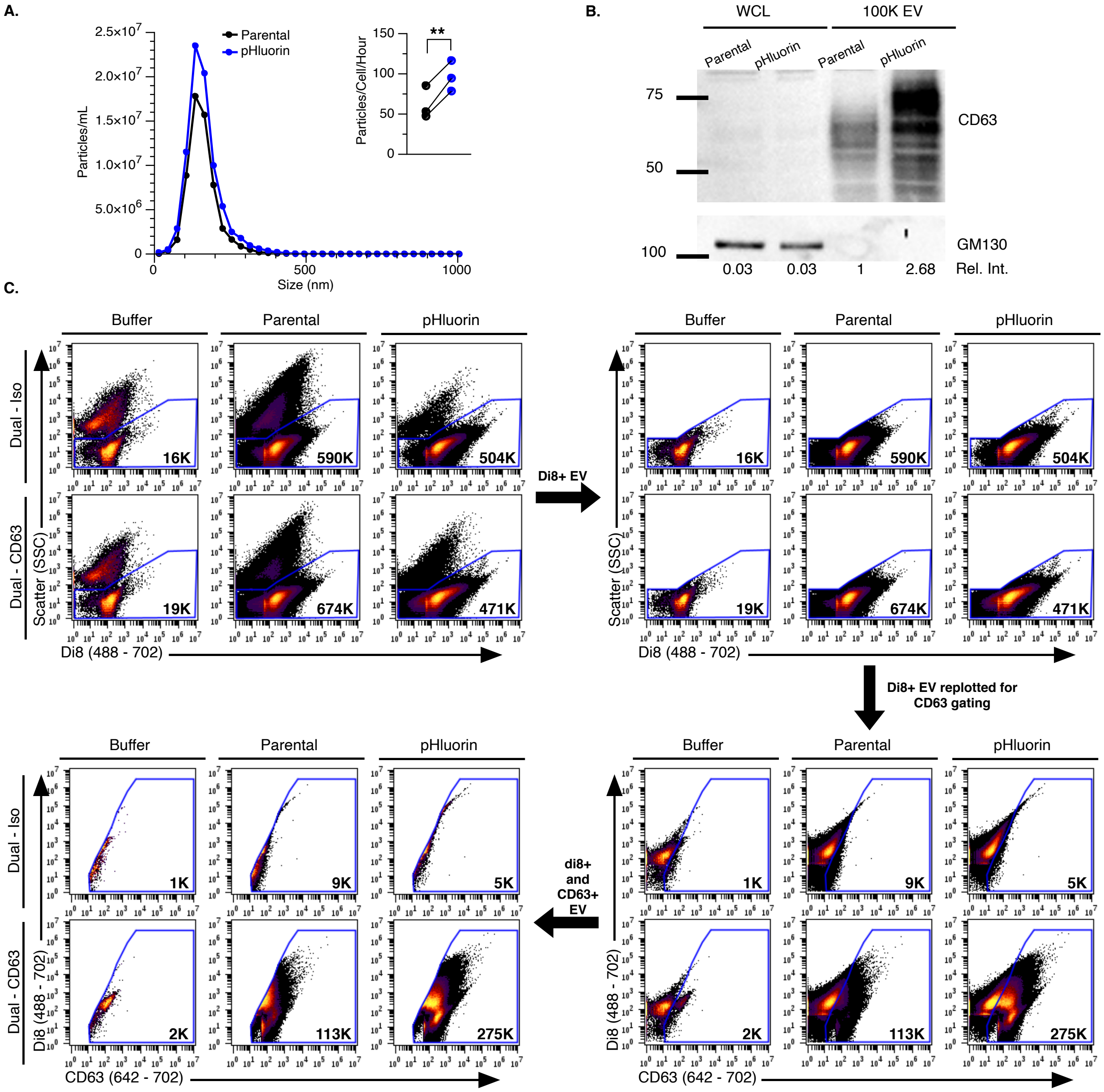

Figure S10. (A) Nanoparticle tracking analysis (NTA) showing size distribution and estimated EV secretion rate (EVs/Cell/Hour) for EVs isolated from HT1080-Parental (Parental) and HT1080 pHluorin-CD63 (pHluorin) cells. Representative size distribution from (n = 1) biological replicate. Secretion rates shown for (n = 3) biological replicates. **(B)** Western blotting analysis of CD63 protein expression in Parental and pHluorin whole cell lysates (WCL) and 100K EV. Relative intensity of CD63 signal in EV preps is shown below (Parental:pHluorin). WCL CD63 signal was normalized to the respective GM130 expression. **(C)** Scatter plots with gates defining di8 positive events as EVs and CD63 positive (CD63+) events. Event counts within the blue gates are depicted.

Gating strategy for multiplex analysis of TSPANs

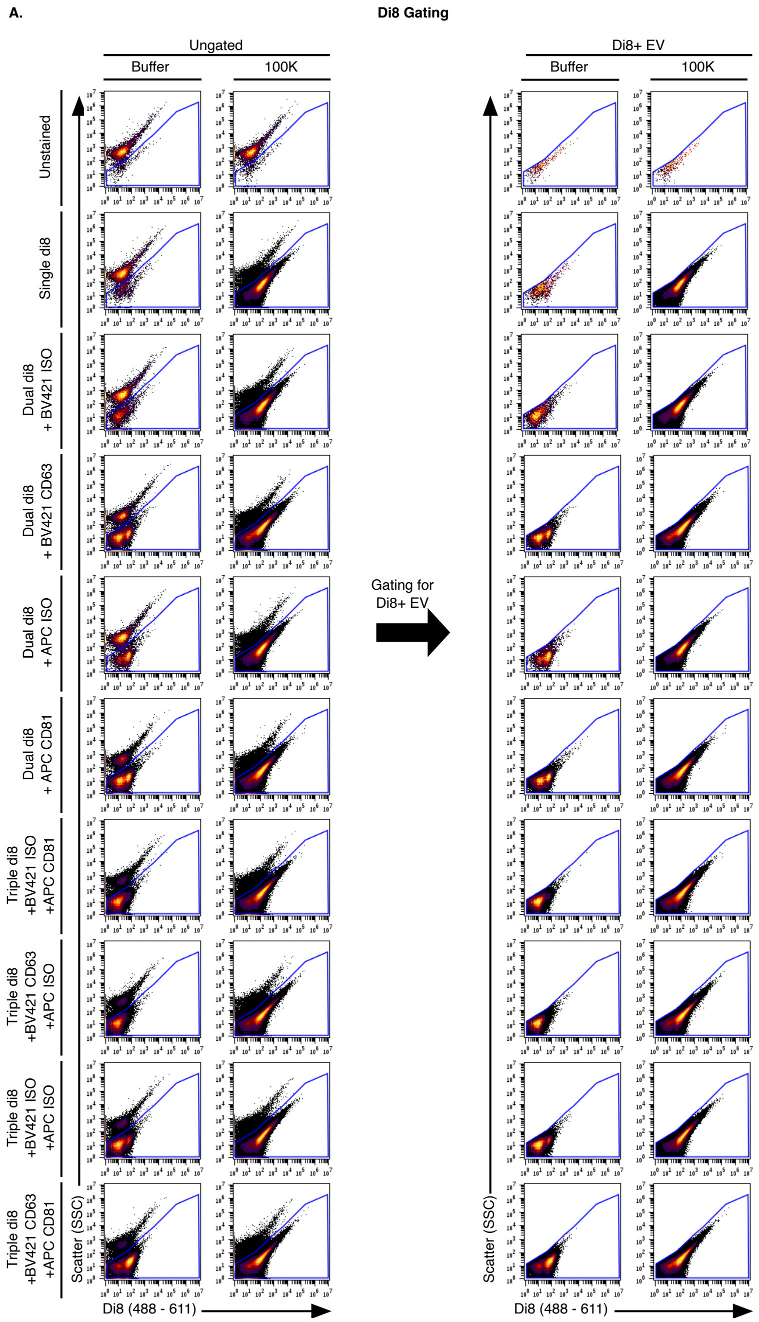

B. **CD63-BV421 Gating**

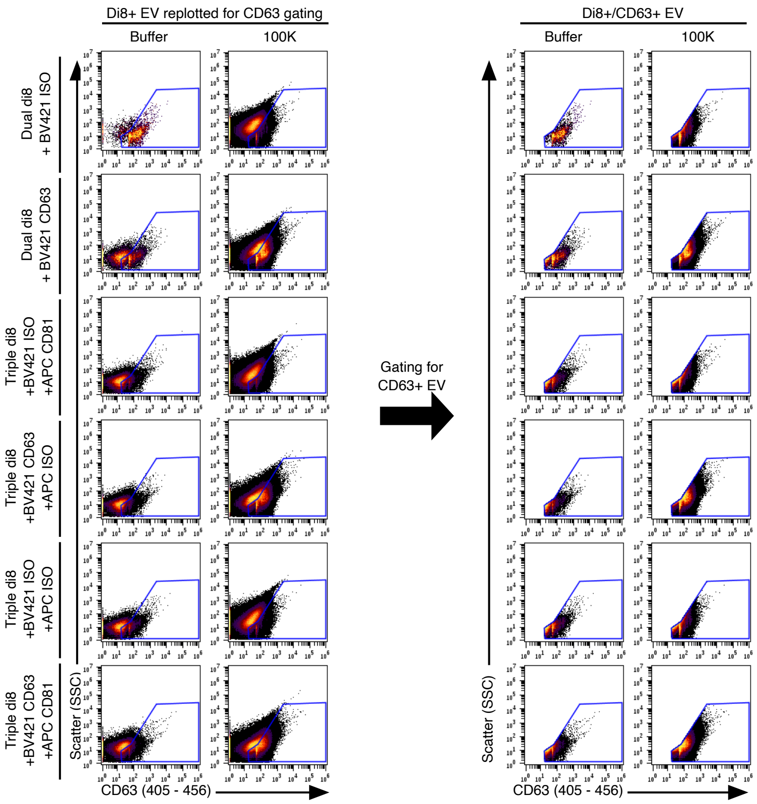

C. **CD81-APC Gating**

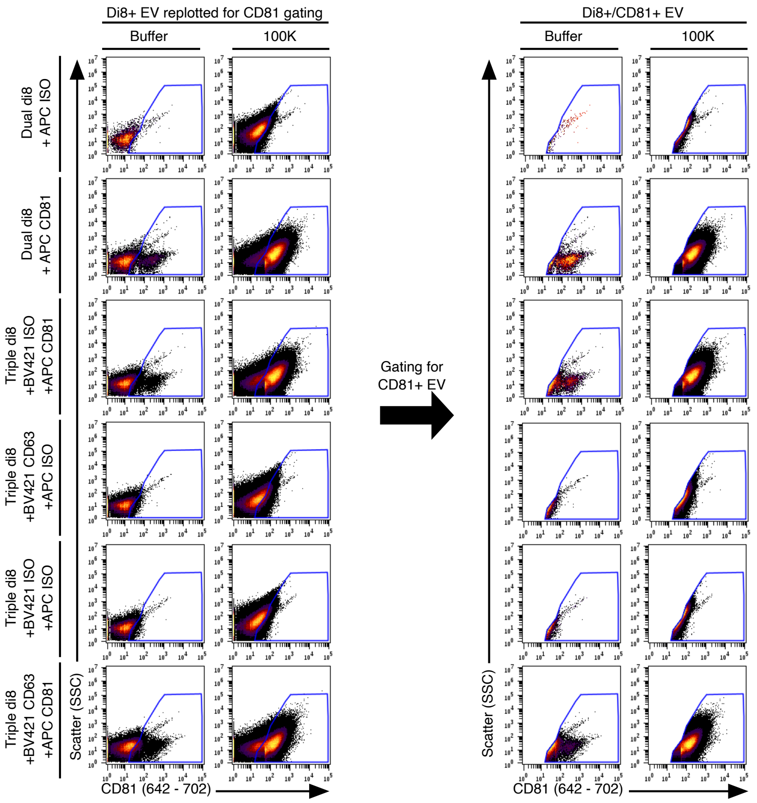

Figure S11. **(A)** Scatter plots with gates defining di8 positive EVs events (EV, 488 - 611) using stained and unstained buffer samples along with unstained 100K EVs as the control. **(B)** Scatter plots of di8 positive EVs from **A** against anti-CD63 BV421 (405 - 456) allowing for selection of CD63+ EVs. **(C)** Scatter plots of di8 positive EVs from **A** against anti-CD81 APC (647-702) allowing for selection of CD81+ EVs.
